# Bacterial crude oil and polyaromatic hydrocarbon degraders from Kazakh oil fields as barley growth support

**DOI:** 10.1007/s00253-024-13010-y

**Published:** 2024-02-02

**Authors:** Kuralay Yessentayeva, Anne Reinhard, Ramza Berzhanova, Togzhan Mukasheva, Tim Urich, Annett Mikolasch

**Affiliations:** 1 Department of Biology and Biotechnology, Al-Farabi Kazakh National University, Al-Farabi Ave 71, 050040 Almaty, Kazakhstan; 2 Institute of Microbiology, University Greifswald, Felix-Hausdorff-Straße 8, 17487 Greifswald, Germany

**Keywords:** Hydrocarbon transformation, *Arthrobacter*, *Bacillus*, *Dietzia*, *Kocuria*, *Micrococcus*

## Abstract

**Abstract:**

Bacterial strains of the genera *Arthrobacter*, *Bacillus*, *Dietzia*, *Kocuria*, and *Micrococcus* were isolated from oil-contaminated soils of the Balgimbaev, Dossor, and Zaburunye oil fields in Kazakhstan. They were selected from 1376 isolated strains based on their unique ability to use crude oil and polyaromatic hydrocarbons (PAHs) as sole source of carbon and energy in growth experiments. The isolated strains degraded a wide range of aliphatic and aromatic components from crude oil to generate a total of 170 acid metabolites. Eight metabolites were detected during the degradation of anthracene and of phenanthrene, two of which led to the description of a new degradation pathway. The selected bacterial strains *Arthrobacter bussei/agilis* SBUG 2290, *Bacillus atrophaeus* SBUG 2291, *Bacillus subtilis* SBUG 2285, *Dietzia kunjamensis* SBUG 2289, *Kocuria rosea* SBUG 2287, *Kocuria polaris* SBUG 2288, and *Micrococcus luteus* SBUG 2286 promoted the growth of barley shoots and roots in oil-contaminated soil, demonstrating the enormous potential of isolatable and cultivable soil bacteria in soil remediation.

**Key points:**

*• Special powerful bacterial strains as potential crude oil and PAH degraders.*

*• Growth on crude oil or PAHs as sole source of carbon and energy.*

*• Bacterial support of barley growth as resource for soil remediation.*

**Supplementary Information:**

The online version contains supplementary material available at 10.1007/s00253-024-13010-y.

## Introduction

The daily production of crude oil in Kazakhstan is 1903 BBL/D/1 K as of March 2023. Kazakhstan is thus the 12th largest oil producer in the world and the 7th in Asia (Trading economics 2023). In 2022, 84.2 million tons of oil were recovered, and 64.3 million tons were exported (Staff report in business, The Astana times 2022). Production of 90.5 million tons is planned for 2023 with the export of 71 million tons (Interfax international information group 2022). This makes Kazakhstan the ninth most important petroleum exporting country (Twin 2022). The oil and gas industry plays an important role in the economic development of Kazakhstan and is one of the main drivers of gross domestic product (GDP) (Tanasheva et al. 2022). Kazakhstan’s crude oil comes mainly from the Tengiz field, which produced 29.2 million tons of oil in 2022, the Kashagan field (12.7 million tons), and the Karachaganak field (11.3 million tons) (Interfax international information group 2022). The production and refining of crude oil are frequently associated with the contamination of the oil fields, the transportation routes, and the oil refineries and petroleum processing plants (Holstein et al. 2018; Issayeva et al. 2023; Yermenbay et al. 2020). To counter these problems and to comply with international environmental standards, regulations have been established (UNECE 2019; WECOOP 2021), and these include remediation measures. There are several ways to clean up crude-oil-contaminated land. One of these is to exploit oil-degrading microorganisms isolated from the contaminated soils. For this purpose, we have isolated and taxonomically classified microorganisms from contaminated soil samples in Kazakhstan. We then investigated their catabolic potential and assessed their suitability for use in remediation projects (Mikolasch et al. 2020, 2019, 2015, 2016). Bacteria and fungi are the main groups of soil microbes that play crucial roles in the biodegradation of hydrocarbons in oil-contaminated soils (Cabral et al. 2022; Espinosa-Ortiz et al. 2022; Mahjoubi et al. 2013; Quatrini et al. 2008; Silva et al. 2015). Communities of native microorganisms that are adapted to long-term pollution conditions are particularly important in the degradation of oil contamination. Their characterization can therefore potentially guide bioremediation of contaminated environments. Long-term pollutions result in the accumulation of metabolites of petroleum hydrocarbons, including polycyclic aromatic and aliphatic hydrocarbons, in the soil, and this results in changes in the biodiversity of microorganisms in the soil (Cabral et al. 2022; Imron et al. 2020; Lawniczak et al. 2020). Structural changes in microbial diversity may serve as a basis for the selection of strains active in bioremediation. The purpose of this study was (i) to assess the abundance and diversity of isolatable and cultivable microorganisms in contaminated soils of the oil fields Balgimbaev, Dossor, and Zaburunye in Kazakhstan, (ii) to select powerful oil degraders with a focus on PAH transformation, and (iii) to study the influence of the isolated oil-degrading microorganisms on the growth of barley as a model for bioremediation.

## Materials and methods

### Soil samples

The locations of the sampling sites were determined using the Global Positioning System (GPS), and the soil samples were extensively characterized (Supplementary Table S1). Soil samples were taken with a sterile soil sampler and placed in sterile containers. The samples were stored in a refrigerator (4 °C) until analysis. The soils of the deposits are characterized by a low content of humus (1–2%) and a small thickness of the soil profile. The soils of Zaburunye deposit belong to the type of sodium chloride solonchaks and were named as ZSS1 and ZSS2. The soils of Balgimbaev deposit are of the sulfate–chloride type of salinity and were named as DSS1 and DSS2. The soils of Dossor deposit are characterized by high salinity and were named as BSS1 and BSS2. The residual hydrocarbon content was determined by the weight method after hydrocarbon extraction with chloroform (Lurie 1978): residual hydrocarbon content 1151–1565 mg kg^−1^ soil for ZSS1, 22,560–33,265 mg kg^−1^ soil for ZSS2, 3990–4158 mg kg^−1^ soil for DSS1, 58,770–61589 mg kg^−1^ soil for DSS2, 5127–5752 mg kg^−1^ soil for BSS1, and 68,216–82,554 mg kg^−1^ soil for BSS2.

### Determination of the aerobic culturable soil microbial community

Bacteria and fungi from the six contaminated soil samples (Supplementary Table S1) were enriched on crude oil supplemented with mineral salts medium for bacteria (MSMB) (Hundt et al. 1998) or mineral salts medium for fungi (MSMF) (Awe et al. 2008) according to a previously described method (Mikolasch et al. 2019; Nhi-Cong et al. 2010). After enrichment, fungi were cultured by plating 0.1 mL of the enriched cultures in MSMF on malt agar (MA) plates. Bacteria enriched in MSMB were plated on Luria Bertani (LB) medium (Miller, Amresco, USA) plates. Pure cultures from MSMB were cultivated on LB and from MSMF on MA slants.

A wide variety of microorganisms were isolated from the six soil samples and cultured on different media for characterization. Heterotrophic microorganisms were grown on LB medium (Miller, Amresco, USA). Micromycetes and spore-forming microorganisms were cultured on Sabouraud medium (Carl Roth, Germany). The grown colonies of bacteria and micromycetes were assigned to morphological types. Bacterial isolates were phenotypically characterized and grouped based on cell morphology, Gram reaction, colony morphology, catalase test, endospore formation tests, glucose gas formation, and ammonia formation from arginine (Holt et al. 1994). Physiological tests were carried out, including growth at various temperatures, pH values, and salt concentrations. Biochemical characteristics of isolates, such as sugar fermentation tests, IMViC tests (indole, methyl red, Voges-Proskauer, and citrate), nitrate reduction tests, and urease tests, were conducted (Dworkin 2001). The grown colonies of filamentous fungi and yeasts were divided into macromorphological types. Identification of micromycetes was carried out by classical microbiological methods based on a set of morphological features and culture physiological properties, using modern determinants for the corresponding groups and genera of micromycetes (Kurtzman et al. 2011).

### Study of growth of oil-oxidizing microorganisms

The isolated aerobic culturable microorganisms were tested by a cascade of four screening steps for their growth on crude oil and polyaromatic hydrocarbons.

1. Bacteria and fungi were sown into Petri dishes on the surface of MSMB or MSMF agar on crude oil. A 4.5 × 1.5-cm test tube filled with crude oil and covered with a cotton swab was placed on the inner side of the cup to give a gaseous phase of crude oil hydrocarbons. The extent of microorganism growth on experimental and control plates was compared.
2. Qualitative assessment of microbial ability for oil degradation was performed by growth in liquid mineral medium, to which 30 ml L^−1^ crude oil was added as the only source of carbon and energy. The microorganisms were cultured in flasks containing 250 mL of MSMB for bacteria and MSMF for fungi, aerated on a rotary shaker (220 rpm) at 25.0 °C for 10 days. The flasks were inoculated with a suspension of 2-day-old cultures of microorganisms with an optical density of 0.25.
3. The ability of strains to transform polyaromatics was studied on mineral agar plates as previously described (Kiyohara et al. 1982). After microorganisms were inoculated on MSMB or MSMF agar in Petri dishes, naphthalene (200 mg), anthracene (100 mg), or phenanthrene (100 mg) was added. Naphthalene was dissolved in methanol, while anthracene and phenanthrene were dissolved in diethyl ether and evenly distributed on the surface of the media. Colonies of microorganisms capable of using hydrocarbons formed lumen zones on the opaque plate.
4. Qualitative assessment of microbial ability for PAH degradation was performed in the same way as described in screening step 3, only here PAHs at a final concentration of 0.1 mg mL^−1^ were used as substrates—naphthalene dissolved in methanol and anthracene and phenanthrene dissolved in diethyl ether.

### Identification of oil-degrading bacteria

The Gram characteristics of the strains SBUG 2285, SBUG 2286, SBUG 2287, SBUG 2288, SBUG 2289, SBUG 2290, and SBUG 2291 were determined by the KOH test (Suslow et al. 1982). The oxidative/fermentative test according to Hugh and Leifson (1953) was used to distinguish between aerobic and facultative anaerobic bacteria. Additionally, these bacteria were grown on LB plates for 48 h and chromosomal DNA was isolated by using grown cell material in 20-µL ddH_2_O and the DNeasy PowerSoil Kit (Qiagen, US) according to the manufacturer’s protocol in combination with a Fastprep step by the Fastprep24 instrument (MP Biomedicals, Germany).

Bacterial 16S rRNA gene sequences were amplified from 1-µL DNA extracts as template with the oligonucleotide primers 616 V (AGAGTTTGATYMTGGCTC, 0.5 µM) and 1492R (GGTTACCTTGTTACGACTT, 0.5 µM) as described before for bacteria (Mikolasch et al. 2019). Sanger sequencing of the 16S rRNA genes of the isolated strains SBUG 2285, SBUG 2286, SBUG 2287, SBUG 2288, SBUG 2289, SBUG 2290, and SBUG 2291 was performed by Eurofins Genomics (Germany) with the 616 V and 1492R primers. The resulting forward and reverse sequences were assembled using the program Geneious (Geneious, USA). The 16S rRNA sequences of these strains were compared with the NCBI nr database using the blastn algorithm (Altschul et al. 1990).

### Characterization of growth of selected powerful degraders of crude oil components in the presence of crude oil by dry weight measurement

Cultivation of *Arthrobacter bussei/agilis* SBUG 2290, *Bacillus atrophaeus* SBUG 2291, *Bacillus subtilis* SBUG 2285, *Dietzia kunjamensis* SBUG 2289, *Kocuria rosea* SBUG 2287, *Kocuria polaris* SBUG 2288, and *Micrococcus luteus* SBUG 2286 was carried out in 250-mL flasks containing 50 mL of MSMB supplemented with 1.5 mL of crude oil from the oil field Balgimbaev, Atyrau region, Kazakhstan, as the sole carbon and energy source. Cultures were incubated at 25 °C and 180 rpm. Assays without substrate and with nutrient broth were used as controls. The dry weight of cultures (biomass grams per liter) was determined after 6 and 10 days of growth by filtering the whole cultures through a glass fiber filter (Whatman®, Dassel, Germany) which was then dried for 2 h at 100 °C. All strains able to grow well on crude oil formed inconsistent cell pellets with the crude oil droplets so that the removal of crude oil was not possible.

### Oil degradation experiments of selected powerful degraders of crude oil components

Cultures of *Arthrobacter bussei/agilis* SBUG 2290, *Bacillus atrophaeus* SBUG 2291, *Bacillus subtilis* SBUG 2285, *Dietzia kunjamensis* SBUG 2289, *Kocuria polaris/rosea* SBUG 2287 and SBUG 2288, and *Micrococcus luteus* SBUG 2286 were shaken in 500-mL shake flasks with 100-mL MSMB and 1-mL crude oil (oil field Balgimbaev, Atyrau region, Kazakhstan) at 30 °C and 180 rpm. Assays without oil substrate and without cells and with cells and 1% glucose as sole source of carbon and energy were used as controls.

The entire culture volume of such transformation assays was extracted according to Mikolasch et al. (2015). The extracts were analyzed by gas chromatography–mass spectrometry (GC–MS). The data are reported as means for two separate experiments with replicated batch cultures. The standard deviation in these replicates was no more than 10%.

### Degradation of PAHs of selected powerful degraders of crude oil components

For transformation analyses with PAHs, the bacteria were cultivated for 24 h in shaken 500-mL flasks containing 100-mL MSMB and 1% glucose as growth substrate. Glucose-grown cells were harvested by centrifugation at 6000 × *g* for 5 min and washed twice in sterile MSMB. Cells were resuspended in 5–10 mL of sterile MSMB. Naphthalene was added to sterilized 500-mL Erlenmeyer flasks as a methanol solution to a final concentration of 0.1 mg mL^−1^. Anthracene and phenanthrene dissolved in diethyl ether were added to sterile 500-mL Erlenmeyer flasks to a final concentration of 0.1 mg mL^−1^. After evaporation of the methanol or diethyl ether for 12 h, 100-mL sterile MSMB was added and flasks were shake-incubated for 24 h at 30 °C and 180 rpm to achieve a saturation of naphthalene, anthracene, or phenanthrene in the aqueous phase. The cell suspension was then added to an optical density (A 540 nm) of 3 and the cultures were incubated at 30 °C and 180 rpm. Assays without substrate, with glucose as substrate, and without cells were used as controls.

For structural characterization of transformation products, the reaction was terminated after 7 days of incubation. The whole cultures of such transformation assays were extracted according to Mikolasch et al. (2015). The extracts were analyzed by GC–MS.

### Chemical analysis and identification of products by GC–MS

The crude oil components and the metabolites produced by the selected degraders of crude oil components were detected and quantified by injecting 1 µL of the extracts of the degradation experiments on crude oil and on PAHs into an Agilent gas chromatograph 7890A GC System (Waldbronn, Germany) equipped with a capillary column (Agilent 1901 S-433, 30 m × 250 µm × 0.25 µm, HP-5 ms column) and a mass selective detector 5975C inert XL EI/CI MSD with a quadrupole mass spectrometer. We used the methods described by Mikolasch et al. (2016) for the analyses of extracts by GC–MS (for analysis of extracts after alkaline extraction elution profile I and for the analysis of extracts after acidic extraction elution profile II).

### Microbial inoculation of barley seeds by selected powerful degraders of crude oil components

The ability of the bacteria *Arthrobacter bussei/agilis* SBUG 2290, *Bacillus atrophaeus* SBUG 2291, *Bacillus subtilis* SBUG 2285, *Dietzia kunjamensis* SBUG 2289, *Kocuria rosea* SBUG 2287, *Kocuria polaris* SBUG 2288, and *Micrococcus luteus* SBUG 2286 to support the growth of barley on oil contaminated sand was tested by the barley seed inoculation method (Mikolasch et al. 2016).

### GenBank accession numbers

Bacterial 16S rRNA gene sequences were deposited in GenBank under the accession numbers OR059175, OR059176, OR059177, OR059178, OR059179, OR059180, and OR059181.

## Results

In total, we isolated 1376 strains of microorganisms from soil samples of the oil-contaminated oil fields Balgimbaev, Dossor, and Zaburunye and characterized them by classical microbiological methods up to the genus level (Table 1).

**Table 1:** Number of isolates of the soil samples of oil fields, with which the search for active destructors was carried out

	Balgimbaev		Dossor		Zaburunye	
	Hydrocarbon content in soil samples, mg kg^−1^ soil					
Genus	5127–5752	68,216–82,554	3990–4158	58,770–61,589	1151–1565	22,560–33,265
	BSS1	BSS2	DSS1	DSS2	ZSS1	ZSS2
Prokaryotes						
*Achromobacter*	5	–	9	–	8	11
*Acinetobacter*	13	5	–	–	5	6
*Arthrobacter*	14	21	8	15	18	29
*Bacillus*	25	21	26	27	33	41
*Dietzia*	–	–	4	3	5	4
*Gordonia*	11	9	9	12	7	15
*Microbacterium*	5	7	9	–	5	3
*Micrococcus*	12	14	11	13	21	18
*Mycobacterium*	15	12	11	17	15	21
*Nocardia*	7	6	7	–	8	7
*Ochrobactrum*	9	8	5	–	6	9
*Pseudomonas*	56	45	35	33	42	50
*Rhodococcus*	23	27	24	41	25	29
*Streptomyces*	9	6	3	5	18	9
Sum	204	181	161	166	216	252
Eukaryotes						
*Aspergillus*	4	2	2	–	2	–
*Aureobasidium*	4	4	6	–	4	6
*Candida*	4		6	–	3	5
*Cladosporium*	2	2	–	–	2	–
*Cryptococcus*	–	–	–	–	–	2
*Metarhizium*	2	–	–	–	–	–
*Metschnikowia*	3	–	–	–	2	2
*Mucor*	2	2	–	–	–	–
*Penicillium*	4	2	2	–	4	2
*Pichia*	–	–	–	–	2	–
*Rhodotorula*	8	6	9	13	16	12
*Trichoderma*	2	2	–	–	–	–
*Trichosporon*	6	5	5	9	5	9
Sum	41	25	30	22	40	38
*Total*	Prokaryotes and eukaryotes					
	245	206	191	188	256	290
Strains after screening step 4	–	2	–	2	–	3

On average, about five times as many prokaryotes than eukaryotes were isolated from the soil samples. Among the prokaryotes, Alphaproteobacteria, Betaproteobacteria, Gammaproteobacteria, Actinomycetia, and Bacilli are represented, with Actinomycetia being the most abundant class. Mainly *Rhodotorula* strains (Basidiomycota, Tremellomycetes) but also Ascomycota like Dothideomycetes, Eurotiomycetes, Microbotryomycetes, Saccharomycetes, and Sordariomycetes were detected among the eukaryotes and some Zygomycota as Mucoromycota.

The 1376 isolated microorganisms were screened by a cascade of four screening steps. During screening step 1 — growth on crude oil on mineral agar (MSMB and MSMF) plates — 576 microorganisms, able to grow on crude oil as sole source of carbon and energy, were selected. Then, a second selection step — growth on 3% crude oil in liquid medium—followed, in which 276 strains were selected. After screening for the ability to transform PAHs on agar plates — screening step 3 — 23 promising microorganisms remained for further investigation. After screening step 4 — growth on PAHs in liquid medium — seven of the 1376 isolated strains were selected as powerful degraders of crude oil components and PAHs and named as XVIIB (17B_7), VIIB (7B_6), ID (1D_4), VIID (7D_5), IIZ (2Z_1), IIIZ (3Z_2), and IVZ (4Z_3). The strains XVIIB (17B_7) and VIIB (7B_6) were isolated from highly contaminated soil of the Balgimbaev oil field (BSS2), ID (1D_4) and VIID (7D_5) from highly contaminated Dossor soil (DSS2), and IIZ (2Z_1), IIIZ (3Z_2), and IVZ (4Z_3) from Zaburunye soil (ZSS2) which contained a high concentration of oil (Table 1). These bacterial isolates were assigned as Gram positive and identified by 16S rRNA sequence analysis, characterization of cell morphology and colony appearance, and biochemical analyses and by comparison of these results with literature data on the corresponding bacterial species (Supplementary Table S2). Bacterial 16S rRNA gene sequences were deposited in GenBank under the accession numbers OR059175 for IIZ (2Z_1) — *Arthrobacter* sp. SBUG 2290, OR059176 for XVIIB (17B_7) — *Bacillus atrophaeus* SBUG 2291, OR059177 for VIIB (7B_6) — *Bacillus subtilis* SBUG 2285, OR059178 for ID (1D_4) — *Dietzia kunjamensis* SBUG 2289, OR059179 for IVZ (4Z_3) — *Kocuria rosea* SBUG 2287, OR059180 for IIIZ (3Z_2) — *Kocuria polaris* SBUG 2288, and OR059181 for VIID (7D_5) — *Micrococcus luteus* SBUG 2286. According to Supplementary Table S2, the characterization of *Arthrobacter* sp. SBUG 2290 is very specific for *A. bussei* or *A. agilis*; therefore, the description *Arthrobacter bussei/agilis* will be used further.

### Growth on crude oil by isolated oil component degraders

To determine the potential of *Arthrobacter bussei/agilis* SBUG 2290, *Bacillus atrophaeus* SBUG 2291, *Bacillus subtilis* SBUG 2285, *Dietzia kunjamensis* SBUG 2289, *Kocuria rosea* SBUG 2287, *Kocuria polaris* SBUG 2288, and *Micrococcus luteus* SBUG 2286 for oil degradation, these strains were cultivated on crude oil and analyzed by the dry weight method. This method was used because all strains had grown on crude oil forming inconsistent compartments of cell pellets and oil residues so that the measurement of optical density was not possible. The dry weight of these seven species increased during cultivation in mineral salts medium (MSMB) supplemented with crude oil as the sole source of carbon and energy (Table 2). Dry weight gains on oil were less than on nutrient broth for all strains; nevertheless, for all seven strains, growth on crude oil resulted in significant values after 10 days. The amount of unmetabolized crude oil decreased during incubation with all seven strains. The highest decrease of 41.1% was achieved with *Arthrobacter bussei/agilis* SBUG 2290 and the lowest decrease of 36.4% by *Micrococcus luteus* SBUG 2286 (Table 2).

**Table 2:** Growth experiments with isolated species on 3% (*v*/*v*) oil after cultivation for 6 and 10 days and controls^a)^

Species	Dry weight after growth on crude oil [g L^−1^]		Dry weight of controls [g L^−1^]			Degradation results	
			Growth on nutrient broth		Inoculum	Decrease of crude oil [%]	Production of products^c)^
	6 days	10 days	6 days	10 days	Start point		
*Arthrobacter bussei/agilis* SBUG 2290	1.6 (0.05)^b)^	4.7 (0.14)	2.8 (0.11)	6.7 (0.27)	0.9 (0.01)	41.2	Yes
*Bacillus atrophaeus* SBUG 2291	1.5 (0.08)	4.7 (0.26)	1.8 (0.12)	5.0 (0.09)	0.7 (0.05)	38.8	Yes
*Bacillus subtilis* SBUG 2285	1.7 (0.07)	3.9 (0.17)	1.9 (0.03)	4.4 (0.07)	0.8 (0.03)	36.7	Yes
*Dietzia kunjamensis* SBUG 2289	1.6 (0.06)	4.1 (0.14)	2.2 (0.08)	5.1 (0.19)	0.8 (0.03)	39.1	Yes
*Kocuria polaris* SBUG 2288	1.6 (0.05)	4.2 (0.24)	1.8 (0.05)	4.5 (0.12)	0.8 (0.04)	37.6	Yes
*Kocuria rosea* SBUG 2287	1.5 (0.11)	4.5 (0.34)	2.1 (0.13)	5.2 (0.32)	0.8 (0.01)	36.5	Yes
*Micrococcus luteus* SBUG 2286	2.3 (0.04)	5.3 (0.09)	2.8 (0.05)	6.0 (0.11)	0.8 (0.02)	36.4	Yes

^a^^)^Dry weight of oil controls after 6 days 0.07 (0.02) g L^−1^, after 10 days 0.07 (0.02) g L^−1^

^b)^Standard deviation

^c)^Data presented in Table 4 and Supplementary Tables S3, S4, S5, S6, S7, S8, S9

### Oil component degradation and metabolite production during growth on crude oil by selected powerful degraders

To characterize the transformations of crude oil components, all of the selected strains were incubated in mineral salts medium (MSMB) supplemented with 3% crude oil as the sole carbon source. After incubation for 6 days, the supernatants were acidified to pH 2 and extracted with diethyl ether, residues dissolved in hexane, methylated by diazomethane, and analyzed by GC–MS (Table 3 and 4 and Supplementary Tables S3, S4, S5, S6, S7, S8, S9). A total of 170 metabolites with acid or acid derivative structures were detected for the seven strains in the turnover on crude oil and a huge amount of well-detected oil components were transformed which were detected in extracts after alkaline extraction (pH9) by GC–MS.

**Table 3:**
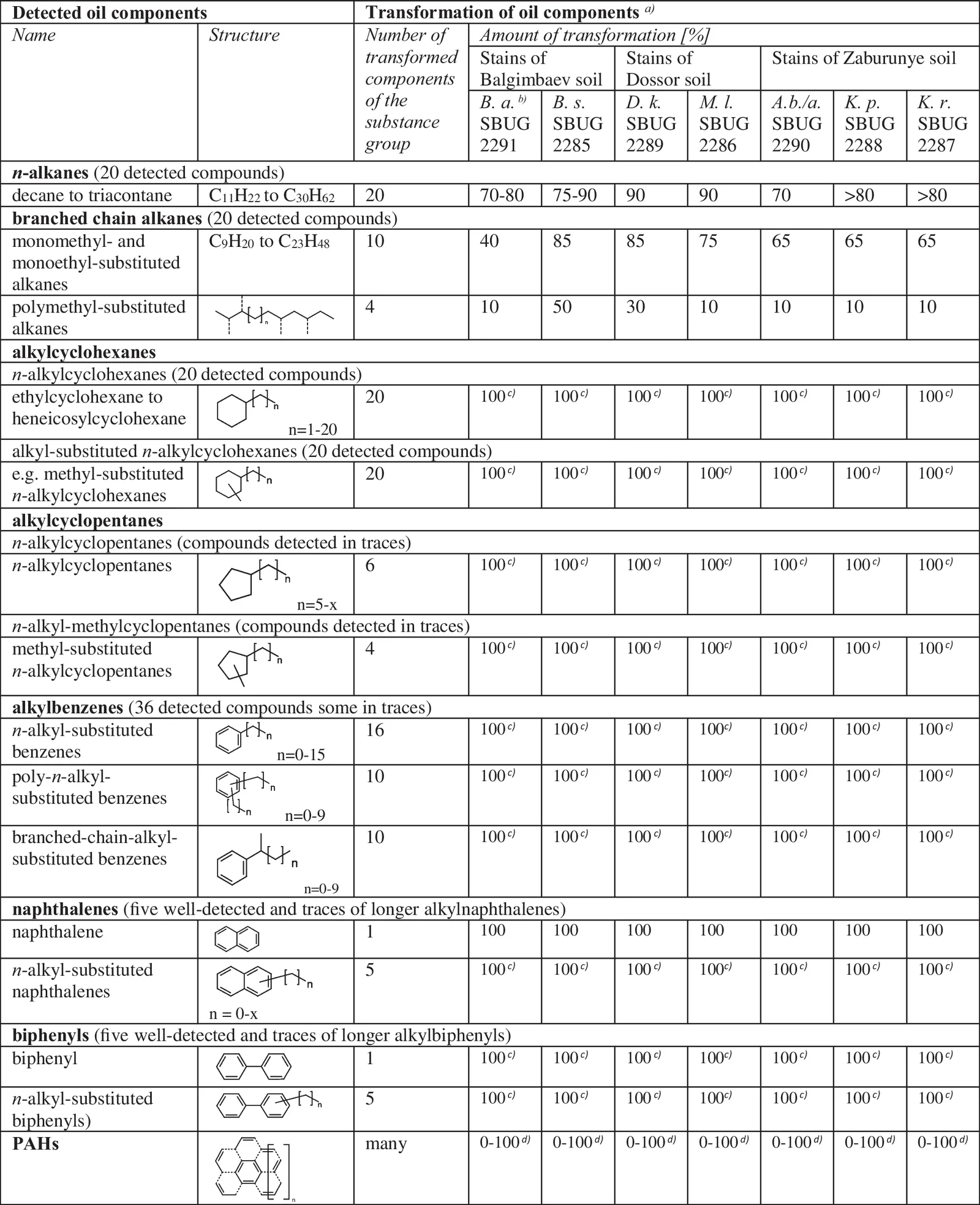
Components of crude oil and their degradation by isolated oil component degraders detected by GC-MS

^*a)*^Not all detected oil components are listed, only the transformation of well-detected substances which could be analyzed in extracts after alkaline extraction (pH9)

^*b)*^*B. a. Bacillus atrophaeus*, *B. s. Bacillus subtilis*, *D. k. Dietzia kunjamensis*, *M. l. Micrococcus luteus*, *A.b./a. Arthrobacter bussei/agilis*, *K. p. Kocuria polaris*, *K. r. Kocuria rosea*

^*c)*^Not detectable after incubation

^*d)*^Amount of transformation depends on the structure

**Table 4:** Aliphatic and aromatic oil components detected by GC–MS and aliphatic and aromatic acids formed during growth of isolated oil component degraders on crude oil

Detected oil components	Corresponding acids formed	Metabolite-producing strain
*Name*	*Name*^*a)*^	
***n*-Alkanes** decane to triacontane		
	**Monoterminal** oxidation product **group A (**Fig. 1**)** in the order of β-oxidation	
Even-numbered	Docosanoic acid M78, M169	*B. atrophaeus* SBUG 2291, *K. polaris* SBUG 2288
	Eicosanoic acid M167	*B. atrophaeus* SBUG 2291
	Tetradecanoic acid M75, M93, M126, M164	*B. atrophaeus* SBUG 2291, *B. subtilis* SBUG 2285, *K. polaris* SBUG 2288, *K. rosea* SBUG 2287
	Dodecanoic acid M91	*K. rosea* SBUG 2287
	Decanoic acid M63, M88, M119	*B. subtilis* SBUG 2285, *K. polaris* SBUG 2288, *K. rosea* SBUG 2287
	Octanoic acid M107	*B. subtilis* SBUG 2285
	Hexanoic acid M97	*B. subtilis* SBUG 2285
Odd-numbered	Tricosanoic acid M79, M170	*B. atrophaeus* SBUG 2291, *K. polaris* SBUG 2288
	Heneicosanoic acid M77, M168	*B. atrophaeus* SBUG 2291, *K. polaris* SBUG 2288
	Nonadecanoic acid M166	*B. atrophaeus* SBUG 2291
	Pentadecanoic acid M94, M165	*B. atrophaeus* SBUG 2291, *K. rosea* SBUG 2287
	Nonanoic acid M57, M147, M160	*B. atrophaeus* SBUG 2291, *K. polaris* SBUG 2288, *M. luteus* SBUG 2286
	Heptanoic acid M80	*K. rosea* SBUG 2287
	Pentanoic acid M95	*B. subtilis* SBUG 2285
	**Diterminal** oxidation product **group B (**Fig. 1**)** in the order of β-oxidation	
Even- and odd-numbered	Decanedioic acid M74	*K. polaris* SBUG 2288
	Nonanedioic acid M71, M124	*B. subtilis* SBUG 2285, *K. polaris* SBUG 2288
	Octanedioic acid M70	*K. polaris* SBUG 2288
	Heptanedioic acid M64	*K. polaris* SBUG 2288
	But-2-enedioic acid M8	*D. kunjamensis* SBUG 2289
	Propanedioic acid M4	*D. kunjamensis* SBUG 2289
	3-Hydroxypropionic acid M1	*D. kunjamensis* SBUG 2289
**Branched chain alkanes**		
	**Monoterminal** oxidation product **group C (**Fig. 1**)**	
Mono(or multiple)-methyl-substituted	12-Methyltetradecanoic acid M76, M128	*B. subtilis* SBUG 2285, *K. polaris* SBUG 2288
	10-Methylundecanoic acid M123	*B. subtilis* SBUG 2285
	2-Methyl-octanoic acid M109	*B. subtilis* SBUG 2285
	2-Methyl-heptanoic acid M104	*B. subtilis* SBUG 2285
	3-Methyl-heptanoic acid M55	*K. polaris* SBUG 2288
	2-Methyl-hexanoic acid M3	*D. kunjamensis* SBUG 2289
	3-Methyl-hexanoic acid M9, M32, M133	*A. bussei/agilis* SBUG 2290, *D. kunjamensis* SBUG 2289, *M. luteus* SBUG 2286
	4-Methyl-hexanoic acid M134	*M. luteus* SBUG 2286
	5-Methyl-hexanoic acid M99	*B. subtilis* SBUG 2285
	3-Methyl-pentanoic acid M132	*M. luteus* SBUG 2286
	4-Methyl-pentanoic acid M96	*B. subtilis* SBUG 2285
	2-Methyl-butanoic acid M131	*M. luteus* SBUG 2286
	3-Methyl-butanoic acid M130	*M. luteus* SBUG 2286
	3-Methyl-alkanoic acid M37, M38, M52, M102, M110, M161	*A. bussei/agilis* SBUG 2290, *B. atrophaeus* SBUG 2291, *B. subtilis* SBUG 2285, *K. polaris* SBUG 2288
	**Diterminal** oxidation product **group D**	
	2-Methyl-propanedioic acid M5	*D. kunjamensis* SBUG 2289
	3-Hydroxy-2-methyl-propionic acid M2	*D. kunjamensis* SBUG 2289
	**Monoterminal** oxidation product **group E (**Fig. 1**)**	
Di(or multiple)-methyl-substituted	3,6-Dimethyl-octanoic acid M86, M115	*B. subtilis* SBUG 2285, *K. rosea* SBUG 2287
	2,6-Dimethyl-heptanoic acid M106	*B. subtilis* SBUG 2285
	2,4-Dimethyl-pentanoic acid M98	*B. subtilis* SBUG 2285
	**Monoterminal** oxidation product **group F (**Fig. 1**)**	
Mono(or multiple)-ethyl-substituted alkanes	2-Ethyl-hexanoic acid M101, M136	*B. subtilis* SBUG 2285, *M. luteus* SBUG 2286
	2-Ethyl-3-oxo-butanoic acid M6	*D. kunjamensis* SBUG 2289
**Alkylcyclohexanes**		
	**Monoterminal** oxidation product **group G (**Fig. 1**)**	
Odd-numbered alkyl substituted cyclohexanes	Cyclohexanecarboxylic acid M10, M53, M81, M103, M138	*B. subtilis* SBUG 2285, *D. kunjamensis* SBUG 2289, *K. polaris* SBUG 2288, *K. rosea* SBUG 2287, *M. luteus* SBUG 2286
	**Alicyclic ring oxidation** product **group H (**Fig. 1**)**	
	1-Cyclohexene-1-carboxylic acid M14, M35, M137	*A. bussei/agilis* SBUG 2290, *D. kunjamensis* SBUG 2289, *M. luteus* SBUG 2286
	**Monoterminal** oxidation product **group I (**Fig. 1**)**	
Even-numbered alkyl substituted cyclohexanes	Cyclohexaneacetic acid M12, M34, M58, M83, M108, M140, M157	*A. bussei/agilis* SBUG 2290, *B. atrophaeus* SBUG 2291, *B. subtilis* SBUG 2285, *D. kunjamensis* SBUG 2289, *K. rosea* SBUG 2287, *K. polaris* SBUG 2288, *M. luteus* SBUG 2286
	**Monoterminal** oxidation product **group J (**Fig. 1**)**	
Methyl-substituted *n*-alkylcyclohexanes	3-Methylcyclohexane-carboxylic acid M56, M139	*K. polaris* SBUG 2288, *M. luteus* SBUG 2286
	4-Methylcyclohexane-carboxylic acid M144	*M. luteus* SBUG 2286
	4-Methylcyclohexaneacetic acid M15, M42, M60	*A. bussei/agilis* SBUG 2290, *D. kunjamensis* SBUG 2289, *K. polaris* SBUG 2288
**Alkylcyclopentanes**		
	**Monoterminal** oxidation product **group K (**Fig. 1**)**	
*n*-Alkylcyclopentanes	Cyclopentaneacetic acid M135	*M. luteus* SBUG 2286
	**Alicyclic ring oxidation** product **group L (**Fig. 1**)**	
	1-Cyclopentene-1-carboxylic acid M7	*D. kunjamensis* SBUG 2289
Methyl-substituted *n*-alkylcyclopentanes	**Monoterminal** oxidation product **group M (**Fig. 1**)**	
	3-Methylcyclopentane-carboxylic acid M100	*B. subtilis* SBUG 2285
**Alkylbenzenes**		
	**Monoterminal** oxidation product **group N (**Fig. 1**)**	
Odd-numbered *n*-alkyl-substituted benzenes	Phenylpropanoic acid M46, M116	*A. bussei/agilis* SBUG 2290, *B. subtilis* SBUG 2285
	Benzoic acid M11, M33, M54, M82, M105, M156 (see also aromatization and ring fission products (Fig. 1))	*A. bussei/agilis* SBUG 2290, *B. atrophaeus* SBUG 2291, *B. subtilis* SBUG 2285, *D. kunjamensis* SBUG 2289, *K. rosea* SBUG 2287, *K. polaris* SBUG 2288
	2-Hydroxybenzoic acid M13, M36, M112 (see also hydroxylation products (Fig. 1))	*A. bussei/agilis* SBUG 2290, *D. kunjamensis* SBUG 2289, *B. subtilis* SBUG 2285
	4-Hydroxybenzoic acid M121 (see also hydroxylation products (Fig. 1))	*B. subtilis* SBUG 2285
	2-Oxo-3-phenylpropanoic acid M163	*B. atrophaeus* SBUG 2291
	**Monoterminal** oxidation product **group O (**Fig. 1**)**	
Even-numbered *n*-alkyl-substituted benzenes	4-Phenylbutanoic acid M51, M68	*A. bussei/agilis* SBUG 2290, *K. polaris* SBUG 2288
	Phenylacetic acid M40, M59, M111, M143, M158 (see also aromatization products (Fig. 1))	*A. bussei/agilis* SBUG 2290, *B. atrophaeus* SBUG 2291, *B. subtilis* SBUG 2285, *K. polaris* SBUG 2288, *M. luteus* SBUG 2286
	2-Hydroxyphenylacetic acid M47, M151 (see also hydroxylation products (Fig. 1))	*A. bussei/agilis* SBUG 2290, *M. luteus* SBUG 2286
	4-Hydroxyphenylacetic acid M25 (see also hydroxylation products (Fig. 1))	*D. kunjamensis* SBUG 2289
	2-Oxo-phenylacetic acid M162	*B. atrophaeus* SBUG 2291
	**Monoterminal** oxidation product **group P (**Fig. 1**)**	
Poly-*n*-alkyl-substituted benzenes	3-(*o*-Tolyl)propanoic acid M69	*K. polaris* SBUG 2288
	3-(*m*-Tolyl)propanoic acid M153	*M. luteus* SBUG 2286
	2-Methylbenzoic acid M39, M142	*A. bussei/agilis* SBUG 2290, *M. luteus* SBUG 2286
	3-Methylbenzoic acid M16, M41, M61, M84, M113, M145	*A. bussei/agilis* SBUG 2290, *B. subtilis* SBUG 2285, *D. kunjamensis* SBUG 2289, *K. rosea* SBUG 2287, *K. polaris* SBUG 2288, *M. luteus* SBUG 2286
	4-Methylbenzoic acid M17, M43, M85, M114, M146, M159	*A. bussei/agilis* SBUG 2290, *B. atrophaeus* SBUG 2291, *B. subtilis* SBUG 2285, *D. kunjamensis* SBUG 2289, *K. rosea* SBUG 2287, *M. luteus* SBUG 2286
	2-Hydroxy-3-methylbenzoic acid M18, M44 (see also hydroxylation products (Fig. 1))	*A. bussei/agilis* SBUG 2290, *D. kunjamensis* SBUG 2289
	2-Hydroxy-4-methylbenzoic acid M19 (see also hydroxylation products (Fig. 1))	*D. kunjamensis* SBUG 2289
	2-Hydroxy-5-methylbenzoic acid M45 (see also hydroxylation products (Fig. 1))	*A. bussei/agilis* SBUG 2290
	4-Ethylbenzoic acid M67, M117	*B. subtilis* SBUG 2285, *K. polaris* SBUG 2288
	3,4-Dimethylbenzoic acid M22, M49, M65, M89, M120, M152	*A. bussei/agilis* SBUG 2290, *B. subtilis* SBUG 2285, *D. kunjamensis* SBUG 2289, *K. rosea* SBUG 2287, *K. polaris* SBUG 2288, *M. luteus* SBUG 2286
	3,5-Dimethylbenzoic acid M21, M48, M87, M118	*A. bussei/agilis* SBUG 2290, *B. subtilis* SBUG 2285, *D. kunjamensis* SBUG 2289, *K. rosea* SBUG 2287
	2,3-Dimethylbenzoic acid M150	*M. luteus* SBUG 2286
	2,5-Dimethylbenzoic acid M149	*M. luteus* SBUG 2286
	Phthalic acid M24, M90, M122	*B. subtilis* SBUG 2285, *D. kunjamensis* SBUG 2289, *K. rosea* SBUG 2287
	Phthalic anhydride M62, M148	*K. polaris* SBUG 2288, *M. luteus* SBUG 2286
	4-Methylphthalic acid M26	*D. kunjamensis* SBUG 2289
	4-Methylphthalic anhydride M154	*M. luteus* SBUG 2286
	3-Methylphthalic anhydride M50	*A. bussei/agilis* SBUG 2290
	4,5-Dimethylphthalic acid M27	*D. kunjamensis* SBUG 2289
Branched-chain-alkyl-substituted benzenes	**Monoterminal** oxidation product **group Q (**Fig. 1**)**	
	2-Phenylpropanoic acid M20, M66	*D. kunjamensis* SBUG 2289, *K. polaris* SBUG 2288
**Alkylnaphthalenes**		
*n*-Alkyl substituted naphthalenes	**Monoterminal** oxidation product **group R (**Fig. 1**)**	
	1-Naphthalenecarboxylic acid M28, M72, M125a	*B. subtilis* SBUG 2285, *D. kunjamensis* SBUG 2289, *K. polaris* SBUG 2288
	2-Naphthalenecarboxylic acid M29, M73, M92, M125	*B. subtilis* SBUG 2285, *D. kunjamensis* SBUG 2289, *K. polaris* SBUG 2288, *K. rosea* SBUG 2287
	1-Naphthaleneacetic acid M30, M127	*B. subtilis* SBUG 2285, *D. kunjamensis* SBUG 2289
Poly-*n*-alkyl-naphthalenes	2,6-Naphthalenedicarboxylic acid M31	*D. kunjamensis* SBUG 2289
	1,8-Naphthalic anhydride M155 (see also ring fission products (Fig. 1))	*M. luteus* SBUG 2286
**Alkylbiphenyls**		
*n*-Alkyl substituted biphenyls	**Monoterminal** oxidation product **group S (**Fig. 1**)**	
	4-Biphenyl-carboxylic acid M129	*B. subtilis* SBUG 2285
**PAHs**		
	**Ring fission** products **(**Fig. 1**)**	
PAHs	Phthalic acid M24, M90, M122	*B. subtilis* SBUG 2285, *D. kunjamensis* SBUG 2289, *K. rosea* SBUG 2287
	Phthalic anhydride M62, M148	*K. polaris* SBUG 2288, *M. luteus* SBUG 2286
	1,8-Naphthalic anhydride M155	*M. luteus* SBUG 2286

^a^^)^See structures of the formed acids in Supplementary Tables S3–S9

#### Selected degraders from the Dossor oil field

**Fig. 1:**
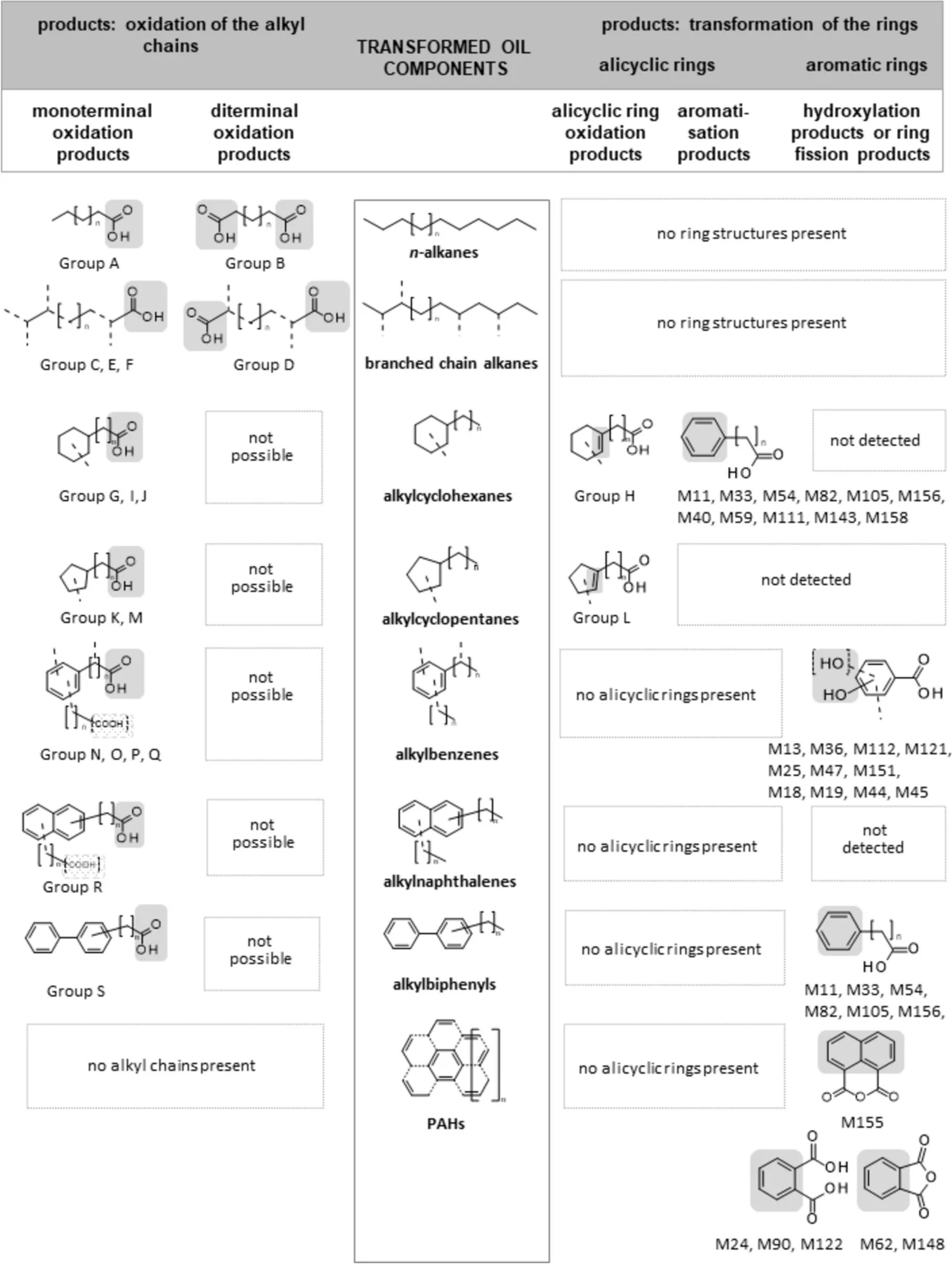
Transformation pathways for the main oil components used by *Arthrobacter bussei/agilis* SBUG 2290, *Bacillus atrophaeus* SBUG 2291, *Bacillus subtilis* SBUG 2285, *Dietzia kunjamensis* SBUG 2289, *Kocuria rosea* SBUG 2287, *Kocuria polaris* SBUG 2288, and *Micrococcus luteus* SBUG 2286

The two species *Dietzia kunjamensis* SBUG 2289 and *Micrococcus luteus* SBUG 2286 isolated from oil-contaminated soil of Dossor deposit were able to degrade aliphatic and aromatic crude oil components (Table 3). *n*-Alkanes with chain length from C_11_ to C_30_ were degraded by 90% and one *omega*-hydroxy monocarboxylic *n*-alkanoic acid — 3-hydroxypropionic acid (M1) — and two dicarboxylic* n*-alkanoic acids — propanedioic acid (M4) and but-2-enedioic acid (M8) — were formed by *Dietzia kunjamensis* SBUG 2289 (Table 4, Fig. 1 diterminal oxidation product group B), whereas only one monocarboxylic *n*-alkanoic acid—nonanoic acid (M147) — was detected for *Micrococcus luteus* SBUG 2286 (Table 4, Fig. 1 monoterminal oxidation product group A). Branched chain alkanes were less well transformed (10–85%) than *n*-alkanes, but more alkanoic acids were detected (Table 3 and 4). While *Dietzia kunjamensis* SBUG 2289 produced three monocarboxylic branched chain alkanoic acids (Table 4, Fig. 1, monoterminal oxidation product group C) and one acid each from *omega*-hydroxy monocarboxylic branched chain alkanoic acids and dicarboxylic branched chain alkanoic acids (Table 4, Fig. 1, diterminal oxidation product group D), six monocarboxylic branched chain alkanoic acids were analyzed from *Micrococcus luteus* SBUG 2286 (Table 4, Fig. 1, monoterminal oxidation product group C, monoterminal oxidation product group F). Only one of the carboxylic branched chain alkanoic acids — 3-methylhexanoic acid (M9, M133) — was recovered from both strains. Therefore, *Dietzia kunjamensis* SBUG 2289 is capable of mono- and diterminal degradation, while *Micrococcus luteus* SBUG 2286 is only capable of monoterminal degradation (Table 4, Fig. 1).

*n*-Alkylcyclopentanes and *n*-hexanes were not detected after incubation with either *Dietzia kunjamensis* SBUG 2289 or *Micrococcus luteus* SBUG 2286, but for both strains, six possible acidic products were detected, with as many as three metabolites occurring in the samples of both strains — cyclohexanecarboxylic acid (M10, M138), cyclohexaneacetic acid (M12, M140), and 1-cyclohexene-1-carboxylic acid (M14, M137), the latter acid is an indication that both strains are capable of alicyclic ring oxidation (Table 4, Fig. 1, alicyclic ring oxidation product group H). The analysis of 1-cyclopentene-1-carboxylic acid (M7, Table 4, Fig. 1, alicyclic ring oxidation product group L) for *Dietzia kunjamensis* SBUG 2289 is a further confirmation of the alicyclic ring oxidation. 1-Cyclohexene-1-carboxylic acid can also be seen as a precursor to the formation of benzoic acid (M11) during the aromatization process (Table 4, Fig. 1, aromatization products), which is a very rarely detected transformation.

*n*-Alkyl-benzenes, poly-*n*-alkylbenzenes, and branched chain alkyl-substituted benzenes were also no longer detected by GC–MS in the supernatants of *Dietzia kunjamensis* SBUG 2289 and *Micrococcus luteus* SBUG 2286 at the end of incubation (Table 3). Thirteen acids — two of the monoterminal oxidation product group N, one of the monoterminal oxidation product group O, nine of the monoterminal oxidation product group P, and one of the monoterminal oxidation product group Q (Table 4, Fig. 1) — were determined for *Dietzia kunjamensis* SBUG 2289, making *Dietzia kunjamensis* SBUG 2289 besides *Kocuria polaris* SBUG 2288 from Zaburunye deposit the only two of the strains analyzed for which a product of branched-chain-alkyl-substituted benzene degradation was detected. Ten acids — two of the monoterminal oxidation product group O and eight of the monoterminal oxidation product group P — were analyzed for *Micrococcus luteus* SBUG 2286. Again, three of these metabolites — 3-methylbenzoic acid (M16, M145), 4-methylbenzoic acid (M17, M146), and 3,4-dimethylbenzoic acid (M22, M152) — were detected in the extracts of both strains. Furthermore, it should be mentioned at this point that these three metabolites were formed by six of the seven strains tested and therefore represent central metabolites in degradation of alkylbenzenes.

*n*-Alkyl-naphthalenes and poly-*n*-alkylnaphthalenes were consumed with the formation of three naphthalene *n*-alkanoic acids (monoterminal oxidation product group R in Table 4, Fig. 1) and one naphthalene *n*-alkanedioic acid — 2,6-naphthalenedicarboxylic acid (M31 also group R) — by *Dietzia kunjamensis* SBUG 2289. 1,8-Naphthalic anhydride (M155 also group R) was the only detectable metabolite formed by *Micrococcus luteus* SBUG 2286, making this strain besides *Dietzia kunjamensis* SBUG 2289 with M31 the only two of the strains analyzed for which a product of poly-*n*-alkyl-naphthalene degradation was detected. Furthermore, M55 can also be a dead-end product of ring fission of PAHs (Table 4, Fig. 1 ring fission products).

*n*-Alkylbiphenyls were metabolized by both strains, but no acidic product was detected, even though both strains showed good transformation rates of these substrates (Table 3). All alkyl-substituted cyclic and aromatic substrates are initially transformed via the monoterminal oxidation pathway of the alkyl chains (Table 4, Fig. 1).

Phthalic acid (M24) and its anhydride (M148) were identified as metabolites of *Dietzia kunjamensis* SBUG 2289 and *Micrococcus luteus* SBUG 2286, respectively, and can be transformation products of PAHs (Table 4, Fig. 1 ring fission products), as for example, anthracene and phenanthrene. The phthalic acid is a product of the ring cleavage of polyaromatic substrates.

#### Selected degraders from Zaburunye deposit

The three strains *Arthrobacter bussei/agilis* SBUG 2290, *Kocuria polaris* SBUG 2288, and *Kocuria rosea* SBUG 2287, isolated from contaminated soil of Zaburunye deposit, degraded crude oil components from different substance groups (Table 3). *n*-Alkanes, chain length from C_11_ to C_30_, were transformed to varying extents — around 70% by *Arthrobacter bussei/agilis* SBUG 2290 and more than 80% by the *Kocuria* species. *Kocuria polaris* SBUG 2288 formed six monocarboxylic *n*-alkanoic acids with chain length of C9 (M57), C10 (M63), C14 (M75), C21 (M77), C22 (M78), and C23 (M79), and *Kocuria rosea* SBUG 2287 secreted five of these acids with chain length of C7 (M80), C10 (M88), C12 (M91), C14 (M93), and C15 (M94) (Table 4, Fig. 1 monoterminal oxidation product group A). Decanoic acid (M63, M88) and tetradecanoic acid (M75, M93) were detected for both *Kocuria* strains. In addition, *Kocuria polaris* SBUG 2288 formed four dicarboxylic *n*-alkanoic acids with chain length of C7 (M64), C8 (M70), C9 (M71), and C10 (M74) (Table 4, Fig. 1 diterminal oxidation product group B). Therefore, *Kocuria polaris* SBUG 2288 is able to degrade *n*-alkanes via mono- and diterminal degradation pathway, while *Arthrobacter bussei/agilis* SBUG 2290 did not appear to secrete any acids of *n*-alkane degradation and therefore no statement on the *n*-alkane degradation path is possible for this strain. Branched chain alkanes were not well utilized (10 to 65% depending from the structure) by *Arthrobacter bussei/agilis* SBUG 2290, *Kocuria polaris* SBUG 2288, or *Kocuria rosea* SBUG 2287. Some monocarboxylic branched chain alkanoic acids were detected including three for *Arthrobacter bussei/agilis* SBUG 2290 (M32, M37, M38) all monoterminal oxidation product group C (Table 4, Fig. 1), but no *omega*-hydroxy monocarboxylic and also no dicarboxylic branched chain alkanoic acids. Overall, it can be deduced from the results of the degradation of *n*- and branched chain alkanes that *Kocuria polaris* SBUG 2288 is capable of mono- and diterminal degradation, while *Arthrobacter bussei/agilis* SBUG 2290 and *Kocuria rosea* SBUG 2287 are only capable of monoterminal degradation.

No products of *n*-alkylcyclopentane degradation were detected for *Arthrobacter bussei/agilis* SBUG 2290, *Kocuria polaris* SBUG 2288, and *Kocuria rosea* SBUG 2287 although *n*-alkylcyclopentanes were not detected after incubation (Table 3 and 4). In contrast, however, two, three, or even four acids were detected in the turnover of alkylcyclohexanes by *Kocuria rosea* SBUG 2287, *Arthrobacter bussei/agilis* SBUG 2290, or *Kocuria polaris* SBUG 2288 belonging to the monoterminal oxidation product groups G, I, and J (Table 4, Fig. 1). Two of these nine cycloalkanoic acids were detected in the extracts of two strains — cyclohexanecarboxylic acid (M53, M81 *K. polaris* SBUG 2288, *K. rosea* SBUG 2287) and 4-methlcyclohexanecarboxylic acid (M42, M60 *A. bussei/agilis* SBUG 2290, *K. polaris* SBUG 2288)—and one was detected for all three strains — cyclohexaneacetic acid (M34, M58, M83). These three acids have also been described for the two strains of Dossor deposit and therefore represent central metabolites in degradation of alkylcyclohexanes.

*n*-Alkyl-benzenes, poly-*n*-alkylbenzenes, and branched chain alkyl-substituted benzenes were also no longer present in the supernatants of *Arthrobacter bussei/agilis* SBUG 2290, *Kocuria polaris* SBUG 2288, and *Kocuria rosea* SBUG 2287. While a total of 28 metabolites—with representatives of all monoterminal oxidation product groups N, O, P, and Q (Table 4, Fig. 1) — were found for the degradation of *n*-alkylbenzenes and poly-*n*-alkylbenzenes, only one intermediate — 2-phenylpropanoic acid (M66) — was detected for the transformation of branched chain alkyl-substituted benzenes by *Kocuria polaris* SBUG 2288 (besides *Dietzia kunjamensis* SBUG 2289 from Dossor deposit the only two of the strains analyzed). Eight poly-*n*-alkylbenzene *n*-alkanoic acids were determined for *Arthrobacter bussei/agilis* SBUG 2290 and five each for the *Kocuria* strains, where again two of these metabolites — 3-methylbenzoic acid (M41, M61, M84) and 3,4-dimethylbenzoic acid (M49, M65, M89) — were detected in the extracts of all three strains and furthermore as mentioned before by six of the seven strains tested and therefore represent central metabolites in degradation alkylbenzenes. Benzoic acid (M33, M54, M82) was also described in the extracts of all three species as product of the *n*-alkyl-benzene degradation and also in the extracts of six of the seven strains tested. Furthermore, benzoic acid can also be a product of aromatization (Table 4, Fig. 1, aromatization products) and can be named as a central metabolite of oil degradation.

*n*-Alkyl-naphthalenes were consumed with the formation of two naphthalene *n*-alkanoic acids — 2-naphthalenecarboxylic acid (M73, M92) by *Kocuria polaris* SBUG 2288 and *Kocuria rosea* SBUG 2287 and 1-naphthalenecarboxylic acid (M72) only by *Kocuria polaris* SBUG 2288 (Table 4, Fig. 1, monoterminal oxidation product group R), whereas none of these acids were detected for *Arthrobacter bussei/agilis* SBUG 2290. Moreover, no corresponding acids were found for the degradation of poly-*n*-alkylnaphthalenes and *n*-alkylbiphenyls by any of these three strains (Table 4), even though all strains showed good transformation rates of these substrates (Table 3). All alkyl-substituted cyclic and aromatic substrates are initially transformed via the monoterminal degradation pathway of the alkyl chains by all three strains (Table 4, Fig. 1).

Phthalic acid anhydride (M62) and phthalic acid (M90) were identified as metabolites of PAH degradation of *Kocuria polaris* SBUG 2288 and *Kocuria rosea* SBUG 2287, respectively. These products are products of the ring cleavage of aromatic substrates (Table 4, Fig. 1 ring fission products).

#### Selected degraders from Balgimbaev deposit

The two species *Bacillus atrophaeus* SBUG 2291 and *Bacillus subtilis* SBUG 2285 isolated from oil-contaminated soil of Balgimbaev deposit were also able to degrade aliphatic and aromatic crude oil components to various extents (Table 3). *n*-Alkanes with chain length from C_11_ to C_30_ were degraded by 70–90%, and eighth and five monocarboxylic* n*-alkanoic acids were measured for *Bacillus atrophaeus* SBUG 2291 and *Bacillus subtilis* SBUG 2285, respectively (Table 4, Fig. 1 monoterminal oxidation product group A). No *omega*-hydroxy monocarboxylic *n*-alkanoic acids were formed by these strains and only one dicarboxylic* n*-alkanoic acid — nonanedioic acid (M124, diterminal oxidation product group B) — was formed by *Bacillus subtilis* SBUG 2285, so that the diterminal degradation pathway could only be detected for *Bacillus subtilis* SBUG 2285 in addition to the monoterminal pathway. Branched chain alkanes were less well transformed by *Bacillus atrophaeus* SBUG 2291 (10–40%) than *n*-alkanes and only one monocarboxylic branched chain alkanoic acid (M161, monoterminal oxidation product group C) was detected, whereas *Bacillus subtilis* SBUG 2285 generated 12 of these acids (8 of monoterminal oxidation product group C, 3 of monoterminal oxidation product group E, 1 of monoterminal oxidation product group F) at transformation rates of 50–85% (Table 3).

Alkylcyclopentanes and hexanes were not detected after incubation of either *Bacillus atrophaeus* SBUG 2291 or *Bacillus subtilis* SBUG 2285. Only the cyclohexaneacetic acid (M157, monoterminal oxidation product group I in Table 4, Fig. 1) was detected as the product for the degradation of *n*-alkylcyclohexanes by *Bacillus atrophaeus* SBUG 2291, while for *Bacillus subtilis* SBUG 2285, both cyclohexanecarboxylic acid (M103, monoterminal oxidation product group G in Table 4, Fig. 1) and cyclohexaneacetic acid (M108) were detected, and for the degradation of alkyl-substituted *n*-alkylcyclopentanes, the 3-methylcyclopentanecarboxylic acid (M100, monoterminal oxidation product group M in Table 4, Fig. 1) was detected.

*n*-Alkyl-benzenes, poly-*n*-alkylbenzenes, and branched chain alkyl-substituted benzenes were also not found in the supernatants of *Bacillus atrophaeus* SBUG 2291 and *Bacillus subtilis* SBUG 2285 at the end of incubation. Five acids belonging to the monoterminal oxidation product groups N, O, and P were determined for *Bacillus atrophaeus* SBUG 2291 and 11 belonging also to the monoterminal oxidation product groups N, O, and P for *Bacillus subtilis* SBUG 2285 (Table 4, Fig. 1), where again three of these metabolites — benzoic acid (M105, M156), phenylacetic acid (M111, M158), and 4-methylbenzoic acid (M114, M159) — were detected in the extracts of both strains. At this point, it should be emphasized once again that these three products represent central intermediates in alkyl-benzene degradation.

Only *n*-alkylnaphthalenes were consumed by *Bacillus subtilis* SBUG 2285 with the formation of three detectable naphthalene *n*-alkanoic acids all monoterminal oxidation product group R, whereas no product of alkylnaphthalene transformation was measured for *Bacillus atrophaeus* SBUG 2291. *n*-Alkylbiphenyls were metabolized by both strains, but 4-biphenyl-carboxylic acid (M129) was the only detected metabolite in the extracts of *Bacillus subtilis* SBUG 2285. Furthermore, M129 was the only detectable metabolite of *n*-alkyl-biphenyl degradation of all seven strains tested.

Only one acid — phthalic acid (M122) — was measured for *Bacillus subtilis* SBUG 2285 in the transformation of PAHs (Table 4), even though both strains showed good transformation rates of PAHs (Table 3 and 5).

For all strains in this study, monoterminal oxidation could be detected for both alkanes and alkyl chains on alicyclic and aromatic hydrocarbons, respectively. With regard to the many monoterminal oxidation products for alkane degradation, it is remarkable that these were only detectable for branched chain alkanes for *Arthrobacter bussei/agilis* SBUG 2290. Diterminal oxidation products were only measured for the strains *Bacillus subtilis* SBUG 2285, *Dietzia kunjamensis* SBUG 2289, and *Kocuria polaris* SBUG 2288 and grouped to the diterminal oxidation product groups B and D (Table 4, Fig. 1).

Overall, under the given conditions, the alicyclic and aromatic hydrocarbons were converted more effectively than the alkanes (Table 3) by all of the seven strains tested, which can probably be explained by the fact that selection steps 3 and 4 to find out the seven powerful degraders were carried out with the aromatics naphthalene, anthracene, and phenanthrene. The detected hydroxylation products and ring cleavage products also suggest good utilization of aromatics and PAHs (Fig. 1).

### Growth on and degradation of PAHs of selected powerful degraders of crude oil components

PAHs are components of the crude oil from the Balgimbaev oil field, Atyrau region, Kazakhstan. Several substances of this group such as naphthalene, anthracene, phenanthrene, and alkyl-substituted derivatives and higher condensed systems were detected in the crude oil by GC–MS. To elucidate the potential of *Arthrobacter bussei/agilis* SBUG 2290, *Bacillus atrophaeus* SBUG 2291, *Bacillus subtilis* SBUG 2285, *Dietzia kunjamensis* SBUG 2289, *Kocuria rosea* SBUG 2287, *Kocuria polaris* SBUG 2288, and *Micrococcus luteus* SBUG 2286 for PAH degradation, these strains were grown on the model compounds of PAH degradation naphthalene, anthracene, and phenanthrene by the dry weight measurement method.

The dry weight of all seven cultures increased during cultivation in mineral salts medium (MSMB) supplemented with these PAHs as the sole source of carbon and energy (Table 5).

But while dry weight on all strains grown on nutrient broth nearly doubled after 6 days and more than doubled again after 10 days, dry weight gains on PAHs were less than those on nutrient broth. All strains grew on PAHs (Table 5), and transformation products were identified by GC–MS (Table 6).

**Table 5:** Growth experiments with isolated species on naphthalene, anthracene, and phenanthrene after cultivation for 6 and 10 days and controls

Species	Dry weight after growth on naphthalene [g L^−1^]		Dry weight after growth on anthracene [g L^−1^]		Dry weight after growth on phenanthrene [g L^−1^]		Dry weight of controls [g L^−1^]			Degradation experiments	
							Growth on nutrient broth		Inoculum	Decrease of PAHs [%]^b)^	Production of products^c)^
	6 days	10 days	6 days	10 days	6 days	10 days	6 days	10 days	Start point		
*Arthrobacter bussei/agilis* SBUG 2290	0.7 (0.02)^a)^	1.4 (0.02)	0.7 (0.01)	1.4 (0.01)	0.8 (0.01)	1.5 (0.07)	0.9 (0.02)	1.7 (0.01)	0.5 (0.01)	100	Yes
*Bacillus atrophaeus* SBUG 2291	0.7 (0.02)	0.9 (0.04)	0.8 (0.03)	1.0 (0.02)	0.6 (0.02)	1.0 (0.03)	0.8 (0.01)	1.8 (0.05)	0.4 (0.01)	100	Yes
*Bacillus subtilis* SBUG 2285	0.7 (0.02)	1.4 (0.03)	0.7 (0.03)	1.0 (0.08)	0.8 (0.04)	1.2 (0.04)	0.7 (0.01)	1.6 (0.06)	0.4 (0.02)	100	Yes
*Dietzia kunjamensis* SBUG 2289	0.8 (0.01)	1.6 (0.05)	0.6 (0.01)	1.1 (0.06)	0.7 (0.01)	1.1 (0.05)	0.9 (0.01)	1.8 (0.01)	0.5 (0.01)	100	Yes
*Kocuria polaris* SBUG 2288	0.8 (0.02)	1.6 (0.05)	0.6 (0.02)	1.0 (0.02)	0.7 (0.02)	1.3 (0.04)	0.7 (0.02)	1.6 (0.03)	0.5 (0.01)	100	Yes
*Kocuria rosea* SBUG 2287	1.0 (0.02)	1.3 (0.04)	0.6 (0.01)	0.8 (0.02)	0.8 (0.02)	1.2 (0.01)	0.7 (0.02)	1.6 (0.05)	0.5 (0.01)	100	Yes
*Micrococcus luteus* SBUG 2286	0.8 (0.01)	1.3 (0.02)	0.7 (0.01)	0.8 (0.03)	0.7 (0.02)	1.0 (0.03)	0.7 (0.01)	1.7 (0.02)	0.5 (0.01)	100	Yes

^a^^)^Standard deviation

^b)^Not detectable after incubation

^c)^Data presented in Table 6

**Table 6:**
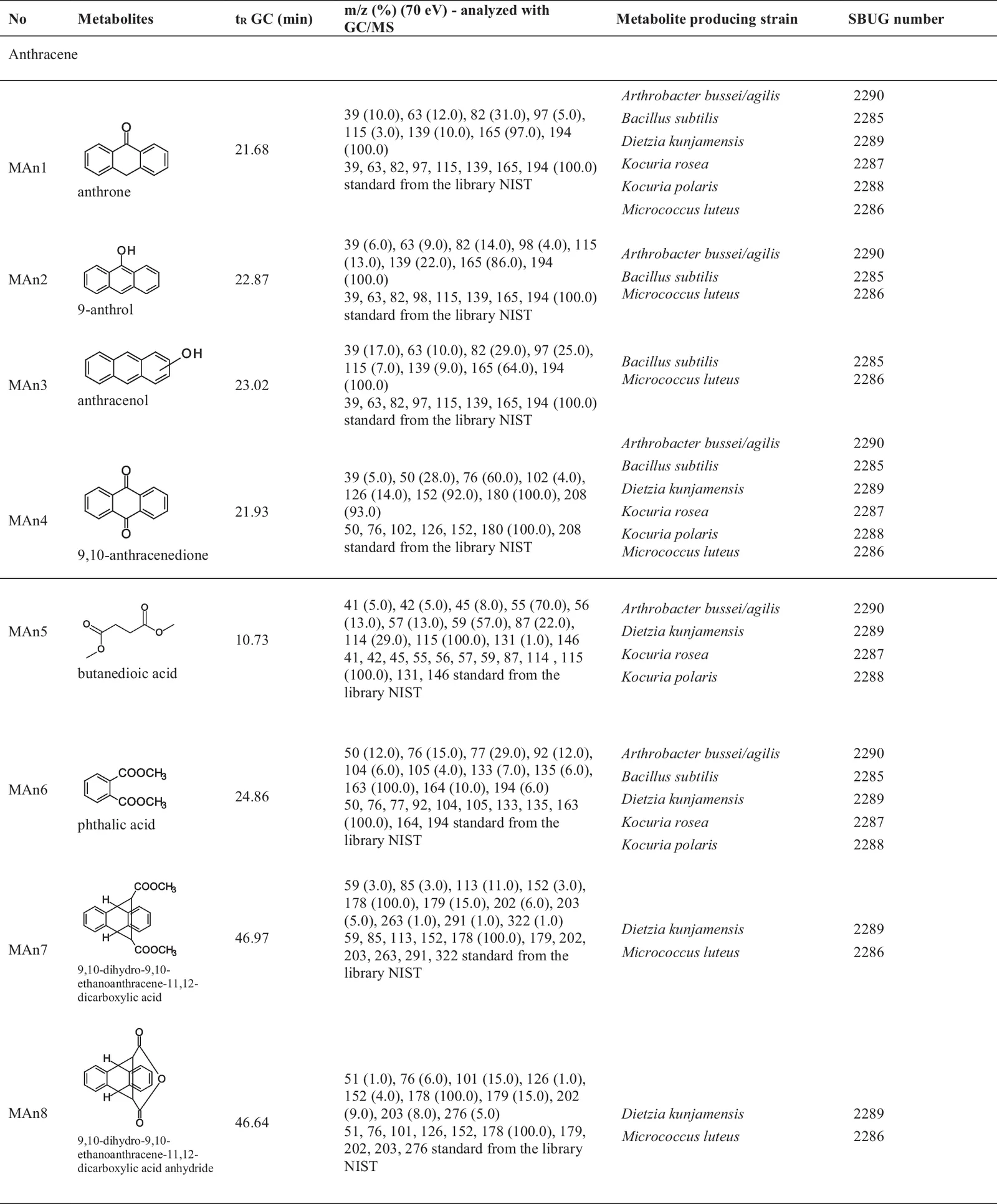

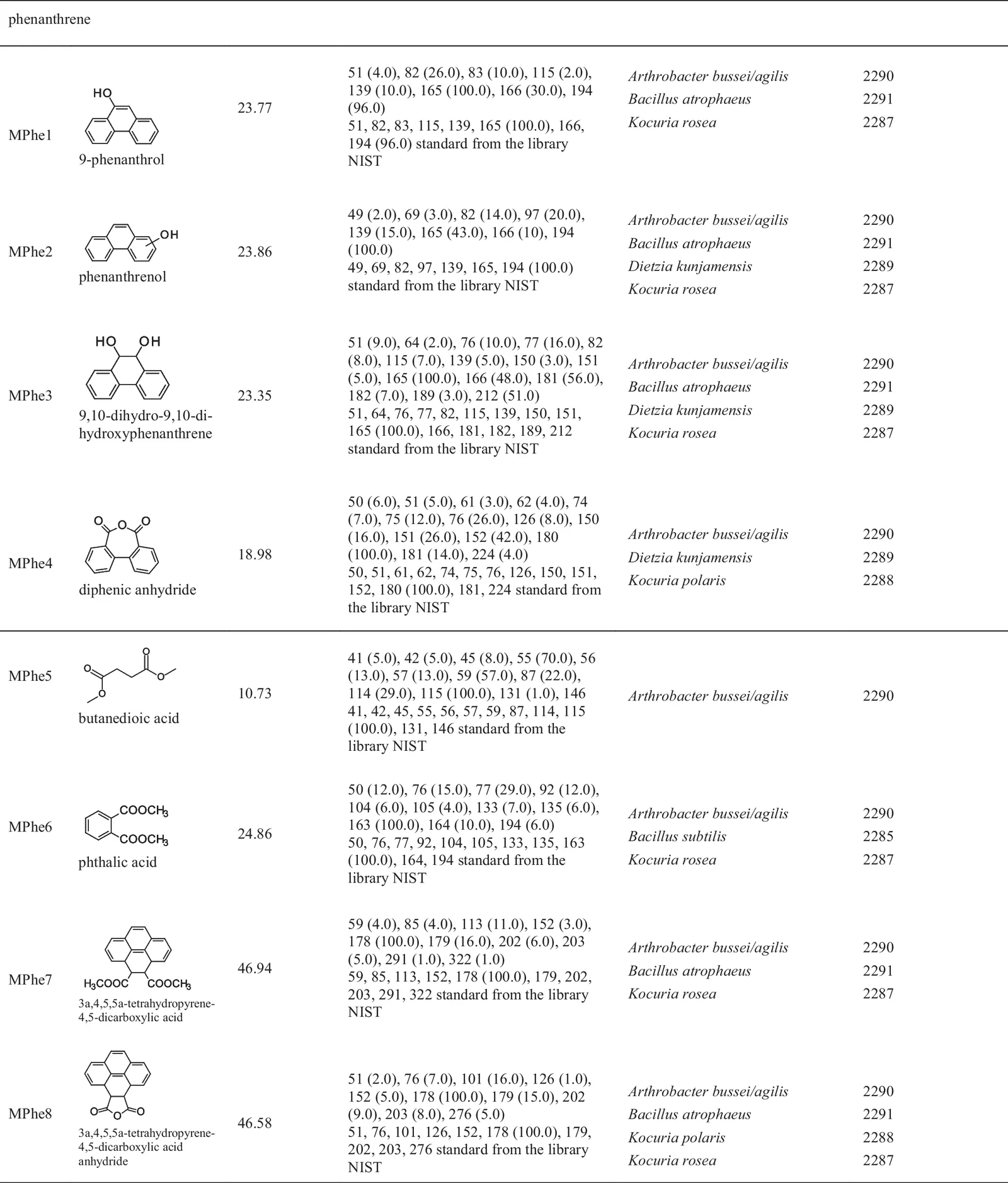
Mass spectrometric data of transformation products identified during incubation on anthracene and phenanthrene. These compounds were analyzed by GC/MS. The extracted acids were transformed for analytical purposes by methylation to the corresponding methyl esters

**Fig. 2:**
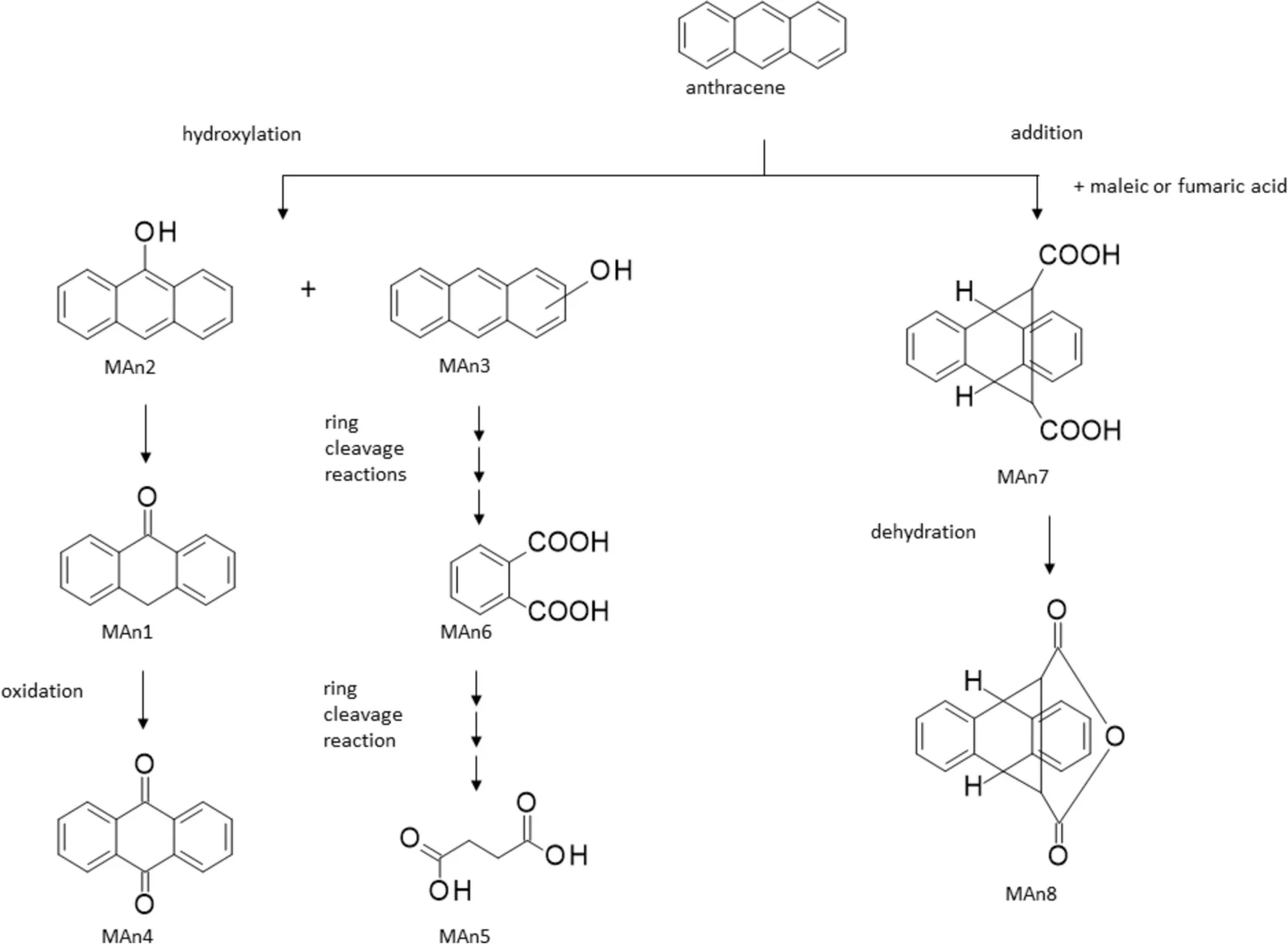
Transformation pathways of anthracene by *Arthrobacter bussei/agilis* SBUG 2290, *Bacillus atrophaeus* SBUG 2291, *Bacillus subtilis* SBUG 2285, *Dietzia kunjamensis* SBUG 2289, *Kocuria rosea* SBUG 2287, *Kocuria polaris* SBUG 2288, and *Micrococcus luteus* SBUG 2286 after 7-day incubation (start OD_540 nm_ = 3) with 0.1 mg mL^−1^ anthracene at 30 °C and 180 rpm and analyses of the whole culture extracts by GC–MS

When *Arthrobacter bussei/agilis* SBUG 2290, *Bacillus subtilis* SBUG 2285, *Dietzia kunjamensis* SBUG 2289, *Kocuria rosea* SBUG 2287, *Kocuria polaris* SBUG 2288, and *Micrococcus luteus* SBUG 2286 were cultured on anthracene, a total of eight different metabolites that might be related to the degradation of anthracene were detected (Table 6), although only two — anthrone (MAn1) and 9,10-anthracenedione (MAn4) — of the eight metabolites were formed by all of these strains. In four products — anthrone (MAn1), 9-anthrol (MAn2), anthracenol (MAn3), and 9,10-anthracenedione (MAn4) — the anthracene structure is still clearly recognizable, since they are hydroxylation and oxidation products, respectively (Fig. 2).

Butanedioic acid (MAn5) and phthalic acid (MAn6) may be products of degradation after ring cleavage and transformation of ring cleavage products have occurred, although no direct ring cleavage products were detected for any of the strains. 9,10-Dihydro-9,10-ethanoanthracene-11,12-dicarboxylic acid (MAn7) and 9,10-dihydro-9,10-ethanoanthracene-11,12-dicarboxylic acid anhydride (MAn8) may be addition products of maleic or fumaric acid to the substrate anthracene and were both measured for *Dietzia kunjamensis* SBUG 2289 and *Micrococcus luteus* SBUG 2286, respectively.

**Fig. 3:**
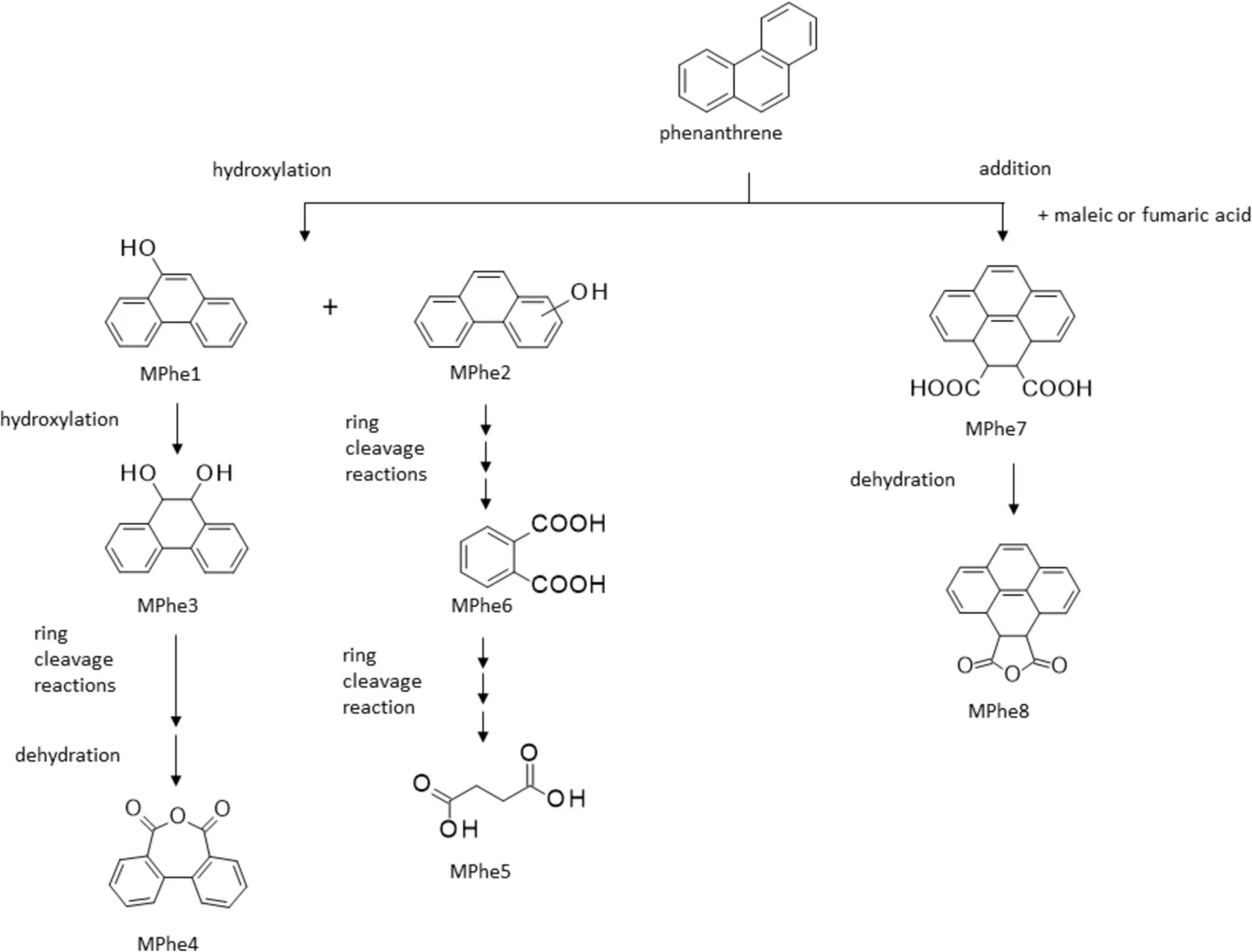
Transformation pathways of phenanthrene by *Arthrobacter bussei/agilis* SBUG 2290, *Bacillus atrophaeus* SBUG 2291, *Bacillus subtilis* SBUG 2285, *Dietzia kunjamensis* SBUG 2289, *Kocuria rosea* SBUG 2287, *Kocuria polaris* SBUG 2288, and *Micrococcus luteus* SBUG 2286 after 7-day incubation (start OD_540 nm_ = 3) with 0.1 mg mL^−1^ phenanthrene at 30 °C and 180 rpm and analyses of the whole culture extracts by GC–MS

After culturing *Arthrobacter bussei/agilis* SBUG 2290, *Bacillus atrophaeus* SBUG 2291, *Bacillus subtilis* SBUG 2285, *Dietzia kunjamensis* SBUG 2289, *Kocuria rosea* SBUG 2287, *Kocuria polaris* SBUG 2288, and *Micrococcus luteus* SBUG 2286 on phenanthrene, eight products that may occur in connection with the transformation of this substrate were measured by GC–MS, and none of these metabolites were detected for all strains. The phenanthrene structure is still recognizable in three products—9-phenanthrol (MPhe1), phenanthrenol (MPhe2), and 9,10-dihydro-9,10-di-hydroxyphenanthrene (MPhe3). Diphenic anhydride (MPe4) is the anhydride of biphenyl-2,2′-dicarboxylic acid, a possible ring cleavage product of phenanthrene (Fig. 3). Other possible degradation products are butanedioic acid (MPhe5) and phthalic acid (MPhe6), which can be formed after further ring cleavages and transformations of these cleavage products. These two products also already appeared as metabolites during the transformation of anthracene as AMAn5 and MAn6. Also, just as in the case of anthracene, two equivalent addition products — 3a,4,5,5a-tetrahydropyrene-4,5-dicarboxylic acid (MPhe7) and 3a,4,5,5a-tetrahydropyrene-4,5-dicarboxylic acid anhydride (MPhe8) — were detected during the conversion of phenanthrene, which could have been formed here by addition of maleic or fumaric acid to the substrate phenanthrene.

### Influence of bacterial inoculation of seeds on the growth behavior of barley

**Fig. 4:**
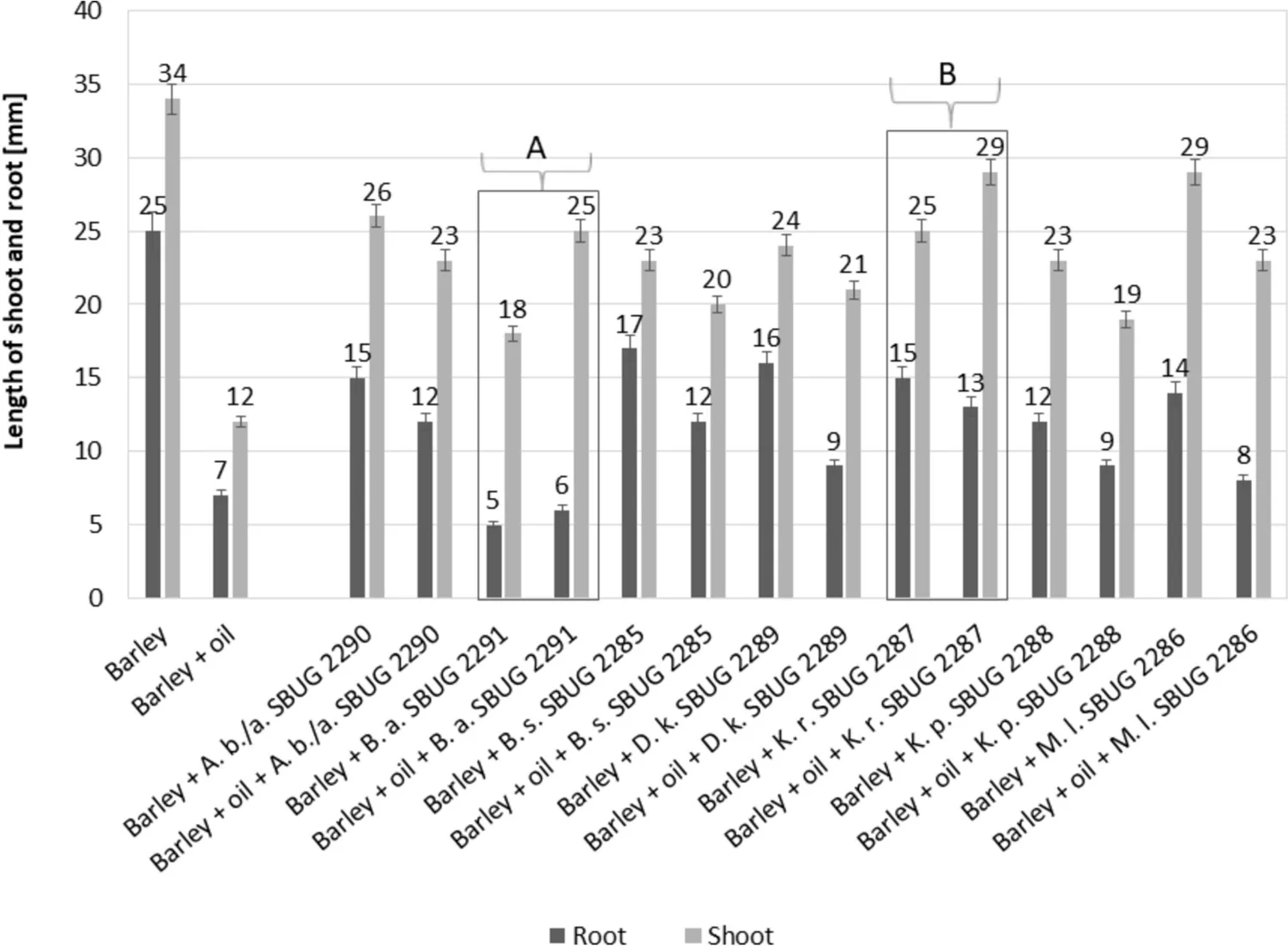
Influence of bacterial inoculation of barley seeds on plant development in oil-containing sand. *Arthrobacter bussei/agilis* SBUG 2290 (A. b. SBUG 2290), *Bacillus atrophaeus* SBUG 2291 (B. a. SBUG 2291), *Bacillus subtilis* SBUG 2285 (B. s. SBUG 2285), *Dietzia kunjamensis* SBUG 2289 (D. k. SBUG 2289), *Kocuria rosea* SBUG 2287 (K. r. SBUG 2287), *Kocuria polaris* SBUG 2288 (K. p. SBUG 2288), *Micrococcus luteus* SBUG 2286 (M. l. SBUG 2286)

To investigate the ability of *Arthrobacter bussei/agilis* SBUG 2290, *Bacillus atrophaeus* SBUG 2291, *Bacillus subtilis* SBUG 2285, *Dietzia kunjamensis* SBUG 2289, *Kocuria rosea* SBUG 2287, *Kocuria polaris* SBUG 2288, and *Micrococcus luteus* SBUG 2286 to support the growth of barley on oil-contaminated soil, these oil-degrading bacterial strains were used to inoculate barley seeds. Barley was chosen because it is known to be significantly more resistant to crude oil compared to other plant species (Mukasheva et al. 2012). The barley seeds inoculated with these strains were sown in oil-containing sand and grown for 10 days. The lengths of shoots and roots were then measured (Fig. 4). The growth of bacterial-inoculated barley was generally worse than the growth without inoculation and without oil (Fig. 4 barley). But on oil-contaminated sand, the shoot growth of barley was generally better after inoculation with any of these strains than without inoculation (Fig. 4 barley + oil) and the root growth was approximately doubled for *Arthrobacter bussei/agilis* SBUG 2290, *Bacillus subtilis* SBUG 2285, and *Kocuria polaris* SBUG 2288. The best growth supporting effect for barley in oil-contaminated sand was observed using *Kocuria rosea* SBUG 2287 as inoculum. Shoot growth increased two and a half times and the root growth doubled compared with the growth in oil-contaminated sand without bacterial inoculation (Fig. 4 barley + oil). Furthermore, the growth of barley with this strain and with *Bacillus atrophaeus* SBUG 2291 is better on contaminated sand than in sand without oil (Fig. 4A, B), suggesting that the oil may be converted particularly effectively by these two strains or that these two strains have some other growth-promoting influence on contaminated sand. Overall, all bacterial strains tested have a growth-promoting influence on barley growth in oil-contaminated sand.

## Discussion

From the six samples of crude oil-contaminated soils collected from the Balgimbaev, Dossor, and Zaburunye oil fields, we isolated 1376 microbial strains from which we selected seven powerful oil component degraders. These belong to the bacterial phyla Firmicutes and Actinomycetota. Two of these seven strains, *Dietzia kunjamensis* and *Kocuria polaris*, have not been extensively described in literature, and isolated pure cultures have not so far been shown to be hydrocarbon degraders, though *Dietzia kunjamensis* has been shown to be present in oily microcosms (Al-Mailem et al. 2017). The present investigation shows that these organisms are powerful crude oil degraders and PAH transformers (Table 2, 3, 4, 5, and 6). *Kocuria polaris* SBUG 2288 transformed the crude oil components *n*-alkanes, branched chain alkanes, *n*-alkylcyclohexanes, *n*-alkyl-benzenes, branched chain alkyl-substituted benzenes, *n*-alkylnaphthalenes, and PAHs, and *Dietzia kunjamensis* SBUG 2289 additionally converted *n*-alkylcyclopentanes. These transformations were shown by the detection of metabolites, by cell growth on these substrates, and by substrate consumption. The species of the genus *Dietzia* are ubiquitous *Actinomycetota* distributed in soil and water. *Dietzia kunjamensis* strain was first isolated from a soil sample from the Indian Himalayas (Mayilraj et al. 2006). Normal- and branched-alkane and alkylcycloalkane degradation has been reported for some other strains of the genus *Dietzia* (Alonso-Gutierrez et al. 2011; Alvarez 2003; Bihari et al. 2010; Chen et al. 2017; Dellagnezze et al. 2014; Gurav et al. 2017; Solyanikova and Golovleva 2019; von der Weid et al. 2007; Wang et al. 2011; Xu et al. 2022; Zoltan et al. 2011). In some cases, the organisms are merely described as *Dietzia* sp., so it is quite conceivable that *Dietzia kunjamensis* strains could be among them.

There are also several reports of *Dietzia* being involved in the degradation of aromatic compounds: carbazole, quinoline, naphthalene, and toluene by *Dietzia cinnamea* (von der Weid et al. 2007); hexacyclic aromatic hydrocarbons by a *Dietzia* sp. (Gurav et al. 2017); phenanthrene and methylphenanthrenes by *Dietzia maris* (Dellagnezze et al. 2014); phenanthrene by *Dietzia psychralcaliphila* (Ausuri et al. 2021); or PAHs by a *Dietzia* sp. (Gerbeth et al. 2004). In view of all these results, our characterization of *Dietzia kunjamensis* as a powerful hydrocarbon degrader may not be surprising, but can now be done for the first time in this study.

*Kocuria polaris* strains have been isolated from or detected in various habitats, e.g., from an Antarctic cyanobacterial mat sample (Reddy et al. 2003), from rhizosphere soils of the Gansu Beishan mountains area (Liu et al. 2013). However, to date, nothing is known about the degradation of petroleum hydrocarbons by this species. Degradation reactions of hydrocarbons have been described for other species from the genus *Kocuria*, especially for the closely related species *Kocuria rosea*. *Kocuria rosea* and *Kocuria marina* (Cui et al. 2022) have been shown to degrade short and mid-long chain petroleum hydrocarbons and PAHs. A microbial consortium of *Kocuria rosea* and *Aspergillus sydowii* (Khandelwal et al. 2022) was shown to enhance the degradation of naphthalene, fluorene, phenanthrene, anthracene, and pyrene in soils, while *Kocuria rosea* and *Kocuria flava* (Ahmed et al. 2010) have been shown to grow on naphthalene, fluoranthene, and phenanthrene. *Kocuria rosea* and *Kocuria palustris* (Lara-Severino et al. 2016) were demonstrated to degrade anthracene. In the light of all this, it is not surprising that our *Kocuria rosea* SBUG 2287 was able to degrade more than 85 crude oil components with the formation of 16 aliphatic and aromatic acids (Table 4). Nor that four products were detected for the transformation of anthracene and six for the transformation of phenanthrene (Table 6). But it must be mentioned again that all the results of *Kocuria polaris* in this study describe it as a hydrocarbon degrader for the first time.

The genus *Kocuria* was separated from the genus *Micrococcus* by phylogenetic and chemotaxonomic analysis in 1995 (Stackebrandt et al. 1995). In this reclassification, only the species *M. luteus* and *M. lylae* remained in the genus *Micrococcus*. In our investigations, a *Micrococcus luteus* was also found as *n*-alkane, branched chain alkane, *n*-alkylcyclopentane and *n*-hexane, *n*-alkyl-benzene, *n*-alkylnaphthalene, and PAH degrader. *Micrococcus luteus* has previously been described as a degrader of *n*-alkanes (Ferrari et al. 2019; Hori and Amund 2000; Tuleva et al. 2009), aromatics and PAHs (Hori and Amund 2000; Olowomofe et al. 2019; Toledo et al. 2006). As early as 1995, Stackebrandt et al. described the close relationship of the genera *Micrococcus* and *Arthrobacter*, and in 2016, Buss reclassified the genus *Arthrobacter*, describing four new genera based on *Arthrobacter*. Our strain *Arthrobacter bussei/agilis* SBUG 2290 is capable of degrading various oil components, which is in line with the large number of reports on degradation by *Arthrobacter* over the last six decades (Casellas et al. 1997; Ghosh and Banerjee 1982; Kallimanis et al. 2009; Kämpfer et al. 1991; Klein et al. 1968; Koronelli and Nesterova 1990; Lors et al. 2004; Margesin et al. 2013; Mullakhanbhai and Bhat 1966; Navarro-Llorens et al. 2008; Peng et al. 2012; Plotnikova et al. 2011; Radwan et al. 1996; Seo et al. 2006; Shi et al. 2014; Stevenson 1967; Ummara et al. 2021; Vandera et al. 2015; Wang et al. 2018; Yano et al. 1971; Yuan et al. 2009) and the close relationship between the two genera.

Two of the seven isolated strains—*Bacillus atrophaeus* SBUG 2291 and *Bacillus subtilis* SBUG 2285—belong to the well-described hydrocarbon degraders *Bacilli*. Strains from this genus have been also isolated from various polluted areas and investigated for degradation abilities for decades (Annweiler et al. 2000b; Baruah et al. 2016; Beskoski et al. 2017; Cerniglia et al. 1984; Chaudhary et al. 2015; Hori and Amund 2000; Kachholz and Rehm 1978; Kämpfer et al. 1991; Khanna et al. 2011; Kiamarsi et al. 2019; Raju et al. 2017; Sezen et al. 2020; Shimura et al. 1999; Wang et al. 2022). The close relationship between *B. atrophaeus* and *B. subtilis* is made very clear by the sp. nov. description of *Bacillus atrophaeus* (Nakamura 1989), the reclassification of *Bacillus subtilis* strains as *Bacillus atrophaeus* (Fritze and Pukall 2001), and the use of *Bacillus subtilis* and *Bacillus atrophaeus* strains in a subcomplex used for profiling bioactive peptides and volatiles (Mülner et al. 2020). The very similar degradation performances of *Bacillus atrophaeus* SBUG 2291 and *Bacillus subtilis* SBUG 2285 in the present work (Table 4 and 6) are also evidence of this close relationship. Both strains have been shown to degrade various hydrocarbons (Hori and Amund 2000; Kachholz and Rehm 1978; Kiamarsi et al. 2019; Mandree et al. 2021; Wang et al. 2022; Zhang et al. 2016).

In previous studies on oil degradation, bacterial strains of genera *Bacillus*, *Gordonia*, *Rhodococcus*, and *Sphingobacterium* were isolated from Kazakh oil-contaminated soils from different deposits (Mikolasch et al. 2015, 2016). The *Gordonia* and *Rhodococcus* species were able to degrade *n*-alkanes and *n*-alkyl- and branched-chain-substituted aromatics, but they were not able to use aromatics or PAHs without alkyl substituents during oil degradation. The *Bacillus* and *Sphingobacterium* species could neither grow on nor transform crude oil as compound mixture, but they were able to use naphthalene. The seven strains of this study *Arthrobacter bussei/agilis* SBUG 2290, *Bacillus atrophaeus* SBUG 2291, *Bacillus subtilis* SBUG 2285, *Dietzia kunjamensis* SBUG 2289, *Kocuria rosea* SBUG 2287, *Kocuria polaris* SBUG 2288, and *Micrococcus luteus* SBUG 2286 were able to grow on crude oil and transform *n*-alkyl, branched-chain, aromatic, and polycyclic aromatic structures during oil degradation.

The formation of the intermediates MAn1 to MAn4 during the transformation of anthracene suggests that all six of the tested strains can form hydroxylation or oxidation products (Table 6, Fig. 2). For *Arthrobacter bussei/agilis* SBUG 2290, *Bacillus subtilis* SBUG 2285, *Dietzia kunjamensis* SBUG 2289, *Kocuria rosea* SBUG 2287, and *Kocuria polaris* SBUG 2288, the ring cleavage products MAn5 or MAn6 were also demonstrated, suggesting that they have the ability to completely degrade anthracene. The transformation results of phenanthrene are comparable to the results of anthracene (Table 6, Fig. 3). Four of the tested strains — *Arthrobacter bussei/agilis* SBUG 2290, *Bacillus atrophaeus* SBUG 2291, *Dietzia kunjamensis* SBUG 2289, and *Kocuria rosea* SBUG 2287 — were able to transform phenanthrene via the hydroxylated products MPhe1, MPhe2, and/or MPhe3. Furthermore, a total of three ring cleavage products — MPhe3, MPhe4, and/or MPhe5 — were identified, though all three were only detected in *Arthrobacter bussei/agilis* SBUG 2290, while for the other strains maximally one of them was detected. Similar patterns of degradation products have been described for anthracene and phenanthrene degradation in general and especially for *Arthrobacter* strains (Baboshin et al. 2005; Plotnikova et al. 2001), *Bacillus* strains (Bibi et al. 2018; Doddamani and Ninnekar 2000; Swaathy et al. 2014), *Dietzia* strains (Ausuri et al. 2021), and also for *Bacillus subtilis* and *Micrococcus luteus* (Hori and Amund 2000), whereas little information about anthracene and phenanthrene degradation in general is available for the genus *Kocuria* (Ahmed et al. 2010; Khandelwal et al. 2022; Lara-Severino et al. 2016; Sakshi et al. 2021; Sakshi et al. 2023). Most reports are restricted to observations of degradation rates, with only occasional reference to oxidation or ring cleavage products or catabolic enzymes.

Very surprising, and to our knowledge not yet described for aerobic degradation, are the products MAn7 and MAn8 of anthracene transformation and MPhe7 and MPhe8 of phenanthrene transformation. In principle, these can be formed by addition of maleic or fumaric acid to the substrates anthracene and phenanthrene, forming MAn7 and MPhe7, followed by dehydration to the products MAn8 and MPhe8. The addition of fumarate to PAHs has been described for the anaerobic degradation of methylnaphthalene (Aitken et al. 2018; Annweiler et al. 2000a; Foght 2008; Meckenstock et al. 2016, 2004; Safinowski et al. 2006), but the fumarate addition under anaerobic conditions is also known for monoaromatic substrates such as toluene, xylenes, and ethylbenzene (Foght 2008; Heider et al. 1998; Kniemeyer et al. 2003; Meckenstock et al. 2016; Spormann and Widdel 2000). However, in all these examples, the fumarate binds to aliphatic groups, such as methyl or ethyl groups, and not directly to the aromatic system. Such direct bond formation of fumarate to phenanthrene or anthracene has so far only been demonstrated for photooxidations or Diels–Alder reactions (Creed et al. 1978; Farid et al. 1972; Passarello et al. 2014; Thunberg and Allenmark 2003), but can now be deduced for the first time also for the bacterial aerobic transformation of anthracene by *Dietzia kunjamensis* SBUG 2289 and *Micrococcus luteus* SBUG 2286 (Fig. 2) and of phenanthrene by *Arthrobacter bussei/agilis* SBUG 2290, *Bacillus atrophaeus* SBUG 2291, *Kocuria rosea* SBUG 2287, and *Kocuria polaris* SBUG 2288 (Fig. 3) from the detected metabolites MAn7 and MAn8 of anthracene transformation and MPhe7 and MPhe8 of phenanthrene transformation.

The use of *Arthrobacter bussei/agilis* SBUG 2290, *Bacillus atrophaeus* SBUG 2291, *Bacillus subtilis* SBUG 2285, *Dietzia kunjamensis* SBUG 2289, *Kocuria rosea* SBUG 2287, *Kocuria polaris* SBUG 2288, or *Micrococcus luteus* SBUG 2286 in plant growth experiments resulted in improvements in shoot growth of barley and, with exception of *Bacillus atrophaeus* SBUG 2291, also for improved root growth in the presence of crude oil compared with the growth of non-inoculated seeds. The inoculation of barley with bacteria of the genera *Gordonia* and *Rhodococcus* or with culture mixtures of the genera *Rhodococcus* and *Bacillus* or with the yeast *Moniliella spathulata* promoted the growth of barley in the same way (Mikolasch et al. 2020, 2015, 2016). During the growth on crude oil by the seven strains of this study, 36–41% of the oil was consumed after 10 days (Table 2) so that barley had better chances of development due to the reduced oil content in the sand. Furthermore, during the degradation of the crude oil by the seven powerful oil degraders described here, a large number of acidic products were generated. Root exudates containing organic acids, amino acids, and carbohydrates can create a specific microenvironment in the root zone system (Kumar et al. 2006), can change the pH value of the environment, and can provide optimal conditions for growth of the rhizosphere microbiota (Gerhardt et al. 2009; Kuiper et al. 2004). Given these positive effects, the reduction of the crude oil content and the production of organic acids by *Arthrobacter bussei/agilis* SBUG 2290, *Bacillus atrophaeus* SBUG 2291, *Bacillus subtilis* SBUG 2285, *Dietzia kunjamensis* SBUG 2289, *Kocuria rosea* SBUG 2287, *Kocuria polaris* SBUG 2288, and *Micrococcus luteus* SBUG 2286 in the soil definitely also benefit the development of other microorganisms, and thus, all positive effects together will contribute to the growth increase of barley in planned field studies. In particular, when all seven strains are used together in field experiments, the positive effects multiply together and these strains should contribute in a meaningful way to bioremediation projects in polluted areas such as the oil fields Balgimbaev, Dossor, and Zaburunye.

In conclusion, 1376 strains of microorganisms belonging to 14 different genera of prokaryotes and 13 genera of eukaryotes were isolated from polluted soil of these three oil fields. Seven powerful oil degraders are here identified for the first time. The degradation results of these strains on polluted soils of the oil fields Balgimbaev, Dossor, and Zaburunye demonstrate the enormous potential of microbial performance in the degradation of crude oil components and PAHs and hence for soil remediation in contaminated areas in Kazakhstan. Furthermore, these results indicate that the indigenous microbiome in contaminated soils may itself have a high oil degradation potential and thus the soils should have a so-called self-purification potential.

## Supplementary Information

Below is the link to the electronic supplementary material.

Supplementary file1 (PDF 667 kb)

## Data Availability

Bacterial 16S rRNA gene sequences are stored in the GenBank FASTA format. All required metadata, including collection dates, location, nucleotide sequence, as described in the GenBank data model were provided for each sequence. The authors declare that the data supporting the findings of this study are available within the paper and its Supplementary Information file. Should any raw data files be needed in another format, they are available from the corresponding author upon reasonable request.
