# Supplementary material for: Bacterial crude oil and polyaromatic hydrocarbon degraders from Kazakh oil fields as barley growth support
Source: Appl Microbiol Biotechnol. 2024 Feb 2;108(1):189. doi: 10.1007/s00253-024-13010-y (PMC10837267; doi:10.1007/s00253-024-13010-y)
Supplement: Supplementary file 1 — Supplementary file1 (PDF 667 kb) [file 253_2024_13010_MOESM1_ESM.pdf]

Supplementary Information

Bacterial crude oil and polyaromatic hydrocarbon degraders from Kazakh oil fields as barley growth support

Applied Microbiology and Biotechnology

Kuralay Yessentayeva[2], Anne Reinhard[1], Ramza Berzhanova[2], Togzhan Mukasheva[2], Tim Urich[1], Annett Mikolasch[1]*

[1] University Greifswald, Institute of Microbiology, Felix-Hausdorff-Straße 8, 17487 Greifswald, Germany

[2] Al-Farabi Kazakh National University, Department of Biology and Biotechnology, Al-Farabi Ave 71, 050040 Almaty, Kazakhstan

* Corresponding author

Phone: ++49-3834-420-5917

Fax: ++49-3834-420-5902

Email: annett.mikolasch@uni-greifswald.de

Supplementary Table S1: Sampling sites and sampling conditions of samples from Dossor field, Zaburunye field and Balgimbaev field (Kazakhstan)

	Zaburunye field		Dossor field		Balgimbaev field	
Sample	ZSS1	ZSS2	DSS1	DSS2	BSS1	BSS2
Region	Zaburunye gas and oil field located in Isatayevsky district of Atyrau region, 170 km west-southwest of Atyrau city		Dossor oil field located in Makat district of Atyrau region, 90 km east of Atyrau city		Balgimbaev oil field belongs to the Isatai district of the Atyrau region, 10 km from the village Akkistau	
Characterisation	Solonchak	Solonchak	Sandy solonchak	Sandy solonchak	Solonchak	Solonchak
Date of sampling	September 2019	September 2019	September 2019	September 2019	September 2019	September 2019
Temperature of area (°C)	19 – 20 °C	19 – 20 °C	19 – 20 °C	19 – 20 °C	19 – 20 °C	19 – 20 °C
Longitude	50.149444	50.149444	52.987500	52.987500	51.014722	51.014722
Latitude	46.766667	46.766667	47.533056	47.533056	47.084167	47.084167
Residual hydrocarbon content (mg/kg soil)	1151 - 1565	22560 - 33265	3990 - 4158	58770 - 61589	5127 - 5752	68216 - 82554

Supplementary Table S2: Results of identification of the isolated bacteria by 16S rRNA gene sequence analysis, KOH test to determine the Gram characteristic and oxidative/ fermentative test, and characterization by cell morphology and colony appearance.

**Isolate/ Genbank accession number**	**Result of identification**	**16S rRNA gene sequence analysis** [a)]						**Gram stain**	**Oxidative/ fermentative and glucose teste**	**Cell morphology and colony appearance**
		Description	*Max score*	*Total score*	*Query cover*	*Ident*	*Accession*			
IIZ (2Z_1) SBUG 2290/ OR059175	*Arthrobacter bussei/agilis* determined according to Flegler et al. (2020) and Koch et al. (1995)[a]	*Arthrobacter bussei* strain KR32 16S ribosomal RNA, partial sequence	2608	2608	100%	99.93%	NR_170399.1	gram-positive	OF: not yellow, no evaluation possible G: not yellow, negative	cells: coccus-shaped, red-orange colonies
		Arthrobacter sp. strain JHARAB1_N 16S ribosomal RNA gene, partial sequence	2608	2608	100%	99.93%	MG757950.1			
		Arthrobacter sp. 4_C7_42 16S ribosomal RNA gene, partial sequence	2608	2608	100%	99.93%	EF540497.1			
		Arthrobacter antioxidans strain QL17 chromosome, complete genome	2603	10412	100%	99.86%	CP095501.1			
		Arthrobacter agilis strain G23 16S ribosomal RNA gene, partial sequence	2601	2601	100%	99.86%	MT397140.1			
		Arthrobacter sp. N5 16S ribosomal RNA gene, partial sequence	2601	2601	99%	99.86%	DQ531645.1			
		Arthrobacter sp. VTT E-052904 16S ribosomal RNA gene, complete sequence	2599	2599	100%	99.79%	EF093123.1			
		Arthrobacter agilis strain DSM 20550 16S ribosomal RNA, partial sequence	2597	2597	100%	99.79%	NR_170400.1			
		Arthrobacter agilis strain NCTC2676_1 genome assembly, chromosome: 1	2597	5195	100%	99.79%	LR131272.1			
		Arthrobacter cheniae 16S ribosomal RNA gene, complete sequence	2597	2597	100%	99.79%	JX949321.2			
XVIIB (17B_7) SBUG 2291/ OR059176	*Bacillus atrophaeus*	Bacillus atrophaeus strain cqs2 16S ribosomal RNA gene, partial sequence	2643	2643	100%	100.00%	MN826517.1	gram-positive	OF: not yellow, no evaluation possible G: yellow, positive	cells: rod-shaped, pre-dominantly solitary rods, oval endospores, dark beige colonies
		Bacillus atrophaeus strain DX1708 16S ribosomal RNA gene, partial sequence	2643	2643	100%	100.00%	MT250840.1			
		Bacillus atrophaeus strain SCA-164 16S ribosomal RNA gene, partial sequence	2643	2643	100%	100.00%	MT125703.1			

		Bacillus atrophaeus strain EGI76 16S ribosomal RNA gene, partial sequence	2643	2643	100%	100.00%	MN704540.1			
		Bacillus atrophaeus strain LKYLW-1 16S ribosomal RNA gene, partial sequence	2643	2643	100%	100.00%	MF375905.1			
		Bacillus atrophaeus strain AKLSR1 16S ribosomal RNA gene, partial sequence	2643	2643	100%	100.00%	MG920142.1			
		Bacillus velezensis strain C40101 16S ribosomal RNA gene, partial sequence	2643	2643	100%	100.00%	MK070087.1			
		Bacillus sp. (in: Bacteria) strain Z41 16S ribosomal RNA gene, partial sequence	2643	2643	100%	100.00%	MG470693.1			
		Bacillus atrophaeus strain JMB002 16S ribosomal RNA gene, partial sequence	2643	2643	100%	100.00%	MG546212.1			
		Bacillus atrophaeus strain BA59 chromosome, complete genome	2643	22878	100%	100.00%	CP024051.1			
VIIB (7B_6) SBUG 2285/ OR059177	*Bacillus subtilis*	*Bacillus subtilis* strain At3 chromosome, complete genome	2643	29030	100%	100.00%	CP051462.1	gram-positive	OF: not yellow, no evaluation possible G: yellow, positive	cells: rod-shaped, single, in pairs and in short chains, oval endospores, beige-coloured colonies
		Bacillus subtilis subsp. inaquosorum strain T1 16S ribosomal RNA gene, partial sequence	2643	2643	100%	100.00%	MT339257.1			
		Bacillus subtilis strain LSRBMoFPIKRGCFTRI24 16S ribosomal RNA gene, partial sequence	2643	2643	100%	100.00%	MT133331.1			
		Bacillus sp. (in: Bacteria) strain 74 16S ribosomal RNA gene, partial sequence	2643	2643	100%	100.00%	MT126330.1			
		Bacillus sp. (in: Bacteria) strain M14 16S ribosomal RNA gene, partial sequence	2643	2643	100%	100.00%	MN714635.1			
		Bacillus subtilis strain G16 16S ribosomal RNA gene, partial sequence	2643	2643	100%	100.00%	MK049903.1			
		Bacillus subtilis subsp. inaquosorum strain AHV-KH11 16S ribosomal RNA gene, partial sequence	2643	2643	100%	100.00%	MH819659.1			
		Bacterium strain BLE9.4 16S ribosomal RNA gene, partial sequence	2643	2643	100%	100.00%	MN044963.1			

		Bacillus subtilis strain 810SS1 16S ribosomal RNA gene, partial sequence	2643	2643	100%	100.00%	MK713698.1			
		Bacillus subtilis strain cd4 16S ribosomal RNA gene, partial sequence	2643	2643	100%	100.00%	MK256680.1			
ID (1D_4) SBUG 2289/ OR059178	*Dietzia kunjamensis* determined according to Mayilraj et al. (2006)[a]	*Dietzia maris* 16S ribosomal RNA gene, partial sequence	2571	2571	99%	99.93%	MG195153.1	gram-positive	OF: not yellow, no evaluation possible G: not yellow, negative	cells: rod-shaped and coccoid, single and in groups, red colonies
		Dietzia sp. K1D201 16S ribosomal RNA gene, partial sequence	2571	2571	99%	99.93%	KX097064.1			
		Dietzia maris strain IHBB 9296 16S ribosomal RNA gene, partial sequence	2571	2571	99%	99.93%	KR085794.1			
		Dietzia kunjamensis strain 313 chromosome, complete genome	2571	7709	99%	99.93%	CP099712.1			
		Dietzia kunjamensis strain D143 16S ribosomal RNA gene, partial sequence	2571	2571	99%	99.93%	ON810444.1			
		Uncultured bacterium clone 24B33 16S ribosomal RNA gene, partial sequence	2571	2571	99%	99.93%	JN882174.1			
		Dietzia sp. WR-3 gene for 16S rRNA, partial sequence	2571	2571	99%	99.93%	AB576128.1			
		Dietzia sp. f18(2011) 16S ribosomal RNA gene, partial sequence	2571	2571	99%	99.93%	HQ652543.1			
		Dietzia sp. f5(2011) 16S ribosomal RNA gene, partial sequence	2571	2571	99%	99.93%	HQ652542.1			
		Dietzia kunjamensis strain DSM 44907 16S ribosomal RNA, partial sequence	2571	2571	99%	99.93%	NR_116684.1			
IVZ (4Z_3) SBUG 2287/ OR059179	*Kocuria rosea* determined according to Stackebrandt et al. (1995)[a]	*Kocuria* sp. strain HY2 16S ribosomal RNA gene, partial sequence	2590	2590	100%	99.86%	MT568619.1	gram-positive	OF: not yellow, no evaluation possible G: not yellow, negative	cells: coccoid, single, in pairs and in clusters, yellow-orange colonies
		Uncultured bacterium clone Untreatedsoil-0day-54 16S ribosomal RNA gene, partial sequence	2590	2590	100%	99.86%	MF314777.2			
		Kocuria rosea strain RR75 16S ribosomal RNA gene, partial sequence	2584	2584	100%	99.79%	MK532258.1			
		Kocuria himachalensis gene for 16S ribosomal RNA, partial sequence, strain: JCM 13326	2584	2584	100%	99.79%	LC113906.1			
		Kocuria rosea strain IHBB 9835 16S ribosomal RNA gene, partial sequence	2584	2584	100%	99.79%	KR085904.1			

		Kocuria rosea strain IHBB 11066 16S ribosomal RNA gene, partial sequence	2584	2584	100%	99.79%	KR085855.1			
		Kocuria sp. BSw21858 16S ribosomal RNA gene, partial sequence	2584	2584	100%	99.79%	JQ069960.1			
		Kocuria rosea strain 2P03AA 16S ribosomal RNA gene, partial sequence	2584	2584	100%	99.79%	EU977667.1			
		Kocuria sp. S26-8 16S ribosomal RNA gene, partial sequence	2584	2584	100%	99.79%	DQ060377.1			
		Kocuria sp. strain JSM 1684078 16S ribosomal RNA gene, partial sequence	2582	2582	100%	99.79%	MG893103.1			
IIIZ (3Z_2) SBUG 2288/ OR059180	*Kocuria polaris* determined according to Reddy et al. (2003)[a]	*Kocuria polaris* strain M0504 16S ribosomal RNA gene, partial sequence	2601	2601	100%	100.00%	KF924218.1	gram-positive	OF: not yellow, no evaluation possible G: not yellow, negative	cells: coccoid, single or as diplococci, salmon-orange colonies
		Kocuria rosea strain OD41 16S ribosomal RNA gene, partial sequence	2590	2590	100%	99.86%	MG547927.1			
		Kocuria rosea strain SRCF20 16S ribosomal RNA gene, partial sequence	2590	2590	100%	99.86%	KP326562.1			
		Kocuria polaris strain YJ-S12 16S ribosomal RNA gene, partial sequence	2590	2590	99%	99.93%	KF876845.1			
		Kocuria polaris strain CMS 76or 16S ribosomal RNA gene, partial sequence	2588	2588	100%	99.86%	KX959605.1			
		Actinomycetia bacterium strain Qhu-F128 16S ribosomal RNA gene, partial sequence	2588	2588	100%	99.86%	OP881659.1			
		Kocuria polaris strain D48 16S ribosomal RNA gene, partial sequence	2588	2588	100%	99.86%	ON810396.1			
		Kocuria sp. NO19 16S ribosomal RNA gene, partial sequence	2588	2588	100%	99.86%	KC200019.1			
		Kocuria sp. strain FKR18-2 16S ribosomal RNA gene, partial sequence	2586	2586	99%	99.86%	MZ905177.1			
		Kocuria sp. A13 16S ribosomal RNA gene, partial sequence	2586	2586	100%	99.79%	EU372960.1			
VIID (7D_5) SBUG 2286/ OR059181	*Micrococcus luteus*	Micrococcus luteus strain H399 16S ribosomal RNA gene, partial sequence	2584	2584	100%	99.86%	MH669309.1	gram-positive	OF: not yellow, no evaluation possible G: not yellow, negative	cells: cocci, present as diplococci, tetrads or larger cell clusters, yellow colonies
		Micrococcus luteus strain NCCP 16831 chromosome, complete genome	2584	5147	100%	99.86%	CP043842.1			
		Micrococcus luteus strain SA211 chromosome, complete genome	2584	5147	100%	99.86%	CP033200.1			
		Micrococcus sp. strain Actino-43 16S ribosomal RNA gene, partial sequence	2584	2584	100%	99.86%	MH671539.1			

		Micrococcus sp. strain LPSUB9 16S ribosomal RNA gene, partial sequence	2584	2584	100%	99.86%	MH412689.1			
		Micrococcus luteus strain DYPSBB RPF YRP01 16S ribosomal RNA gene, partial sequence	2584	2584	100%	99.86%	KT339390.1			
		Micrococcus sp. 3723 16S ribosomal RNA gene, partial sequence	2584	2584	100%	99.86%	KP345967.1			
		Micrococcus sp. SL257 chromosome	2584	5152	100%	99.86%	CP113053.1			
		Micrococcus sp. strain LD141 16S ribosomal RNA gene, partial sequence	2584	2584	100%	99.86%	MW832366.1			
		Micrococcus luteus strain OH4847 16S ribosomal RNA gene, partial sequence	2584	2584	100%	99.86%	KF030221.1			

[a] Reference List

Flegler A, Runzheimer K, Kombeitz V, Manz AT, von Heilborn DH, Etzbach L, Schieber A, Holzl G, Huttel B, Woehle C, Lipski A (2020) *Arthrobacter bussei* sp. nov., a pink-coloured organism isolated from cheese made of cow's milk. Int J Syst Evol Microbiol 70(5):3027-3036 doi:10.1099/ijsem.0.004125

Koch C, Schumann P, Stackebrandt E (1995) Reclassification of *Micrococcus agilis* (Ali-Cohen 1889) to the genus *Arthrobacter* as *Arthrobacter agilis* comb. nov. and emendation of the genus *Arthrobacter*. Int J Syst Bacteriol 45(4):837-839 doi:10.1099/00207713-45-4-837

Mayilraj S, Suresh K, Kroppenstedt RM, Saini HS (2006) *Dietzia kunjamensis* sp nov., isolated from the Indian Himalayas. Int J Syst Evol Microbiol 56:1667-1671 doi:10.1099/ijs.0.64212-0

Reddy GSN, Prakash JSS, Prabahar V, Matsumoto GI, Stackebrandt E, Shivaji S (2003) *Kocuria polaris* sp nov., an orange-pigmented psychrophilic bacterium isolated from an Antarctic cyanobacterial mat sample. Int J Syst Evol Microbiol 53:183-187 doi:10.1099/ijs.0.02336-0

Stackebrandt E, Koch C, Gvozdiak O, Schumann P (1995) Taxonomic dissection of the genus *Micrococcus: Kocuria* gen nov, *Nesterenkonia* gen nov, *Kytococcus* gen nov, *Dermacoccus* gen nov, and *Micrococcus* Cohn 1872 gen emend. Int J Syst Bacteriol 45(4):682-692 doi:10.1099/00207713-45-4-682

Supplementary Table S3. Mass spectrometric data of aliphatic and aromatic acids identified during growth on crude oil of *Dietzia kunjamensis* SBUG 2289. These compounds were analysed by GC/MS. The extracted acids were transformed for analytical purposes by methylation to the corresponding methyl esters, some acids occur in unmethylated form.

No	t_R (min)	Metabolites	m/z (%) (70 eV) - analyzed with GC/MS
M1	4.7	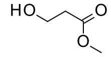	43 (100.0), 59 (12.0), 73 (58.0), 74 (65.0), 103 (3.0)<br>43, 59, 73, 74, 103 standard from the library NIST
M2	6.3	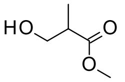	41 (33.0), 57 (65.0), 59 (55), 71 (3.0), 87 (41.0), 88 (100.0), 100 (6.0), 103 (7.0), 119 (1.0)<br>41, 57, 59, 71, 87, 88, 100, 103, 119 standard from the library NIST
M3	7.2	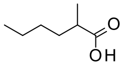	41(31.0), 43 (40.0), 59 (9.0), 74(100.0), 101 (6.0)<br>41, 43, 59, 74, 101 standard from the library NIST
M4	7.3	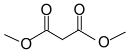	42 (41.0), 43(26.0), 57 (29.0), 59 (100.0), 74 (49.0), 87 (1.0), 101 (93.0), 132 (2.0)<br>42, 43, 57, 59, 74, 87, 101, 132 standard from the library NIST
M5	8.6<br>8.8 st	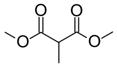	41 (26.0), 43 (31.0), 55 (27.0), 56 (20.0), 57 (46.0), 59 (100.0), 72 (15.0), 87 (21.0), 101 (6.0), 102 (8.0), 114 (14.0), 115 (39.0), 146 (1.0)<br>41 (14.0), 43 (31.0), 55 (22.0), 56 (21.0), 57 (34.0), 59 (100.0), 72 (17.0), 87 (25.0), 101 (9.0), 102 (15.0), 114 (22.0), 115 (59.0), 146 (1.0) measured standard<br>41, 43, 55, 56, 57, 59, 72, 87, 101, 102, 114, 115, 146 standard from the library NIST
M6	9.5	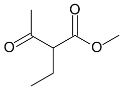	43 (100.0), 55 (27.0), 68 (1.0), 87 (61.0), 102 (32.0), 116 (6.0), 129 (1.0), 144 (1.0)<br>43, 56, 68, 87, 102, 116, 129, 144 standard from the library NIST
M7	10.0<br>10.1 st	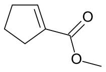	39 (34.0), 41 (45.0), 59 (11.0), 65 (24.0), 66 (28.0), 67 (100.0), 83 (3.0), 95 (49.0), 111 (6.0), 126 (27.0)<br>39 (21.0), 41 (19.0), 59 (7.0), 65 (25.0), 66 (27.0), 67 (100.0), 83 (3.0), 95 (67.0), 111 (10.0), 126 (50.0) measured standard<br>39, 41, 59, 65, 66, 67, 83, 95, 111, 126 standard from the library NIST
M8	10.3<br>10.6 st	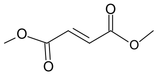	39 (24.0), 53 (36.0),54 (19.0), 59 (48.0), 85 (70.0), 113 (100.0), 114 (21.0), 144 (2.0)<br>39 (1.0), 53 (4.0), 54 (6.0), 59 (16.0), 85 (14.0), 113 (100.0), 114 (8.0), 144 (1.0) measured standard<br>39, 53, 54, 59, 85, 113, 114, 144 standard from the library NIST
M9	11.0	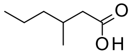	41 (41.0), 42 (21.0), 43 (56.0), 55 (25.0), 60 (100.0), 71 (24.0), 73 (16.0), 87 (34.0), 101 (1.0), 115 (1.0)<br>41, 42, 43, 55, 60, 71, 73, 87, 101, 115 standard from the library NIST

M10	11.6<br>11.9 st	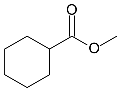	41 (47.0), 55 (100.0), 67 (25.0), 68 (14.0), 69 (10.0), 74 (26.0), 81 (14.0), 82 (22.0), 83 (69.0), 87 (63.0), 101 (9.0), 110 (18,0), 111 (14.0), 113 (21.0), 127 (4.0), 142 (21.0)<br>41 (32.0), 55 (92.0), 67 (21.0), 68 (19.0), 69 (13.0), 74 (39.0), 81 (17.0), 82 (28.0), 83 (81.0), 87 (100.0), 101 (17.0), 110 (31.0), 111 (25.0), 113 (26.0), 127 (10.0), 142 (40.0) measured standard<br>41, 55, 67, 68, 69, 74, 81, 82, 83, 87, 101, 110, 111, 113, 127, 142 standard from the library NIST
M11	12.8<br>13.1 st	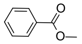	50 (18.0), 51 (37.0), 77 (80.0), 91 (2.0), 92 (3.0), 105 (100.0), 106 (8.0), 136 (30.0)<br>50 (7.0), 51 (17.0), 77 (56.0), 91 (2.0), 92 (2.0), 105 (100.0), 106 (8.0), 136 (38.0) measured standard<br>50, 51, 77, 91, 92, 105, 106, 136 standard from the library NIST
M12	14.8<br>15.1 st	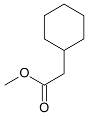	41 (24.0), 43 (34.0), 55 (31.0), 67 (11.0), 74 (100.0), 75 (46.0), 81 (15.0), 97 (6.0), 113 (2.0), 125 (6.0), 156 (1.0)<br>41 (9.0), 43 (15.0), 55 (16.0), 67 (7.0), 74 (100.0), 75 (34.0), 81 (6.0), 97 (6.0), 113 (2.0), 125 (9.0), 156 (1.0) measured standard<br>41, 43, 55, 67, 74, 75, 81, 97, 113, 125, 156 standard from the library NIST
M13	16.3<br>16.5 st	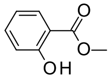	39 (32.0), 63 (23.0), 64 (20.0), 65 (33.0), 92 (84.0), 93 (18.0), 120 (100.0), 121 (31.0), 152 (48.0)<br>39 (12.0), 63 (12.0), 64 (12.0), 65 (19.0), 92 (59.0), 93 (13.0), 120 (100.0), 121 (29.0), 152 (45.0) measured standard<br>39, 63, 64, 65, 92, 93, 120, 121, 152 standard from the library NIST
M14	16.8	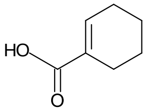	39 (31.0), 41 (30.0), 50 (9.0), 53 (27.0), 55 (17.0), 65 (8.0), 77 (18.0), 79 (54.0), 80 (64.0), 81 (100.0), 108 (24.0), 126 (26.0)<br>39, 41, 50, 53, 55, 65, 77, 79, 80, 81, 108, 126 standard from the library NIST
M15	18.7	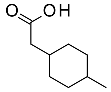	39 (28.0), 41 (54.0), 43 (35.0), 55 (76.0), 60 (37.0), 61 (1.0), 81 (43.0), 96 (26.0), 97 (100.0), 113 (5.0), 156 (1.0)<br>39, 41, 43, 55, 60, 61, 81, 96, 97, 113, 156 standard from the library NIST
M16	19.0	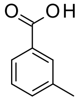	38 (4.0), 51 (8.0), 66 (2.0), 77 (7.0), 89 (11.0), 90 (6.0), 91 (100.0), 92 (17.0), 107 (2.0), 119 (33.0), 136 (51.0)<br>38, 51, 66, 77, 89, 90, 91, 92, 107, 119, 136 standard from the library NIST
M17	19.3	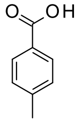	39 (41.0), 50 (9.0), 51 (11.0), 62 (7.0), 65 (28.0), 89 (11.0), 90 (8.0), 91 (100.0), 119 (55.0), 136 (52.0)<br>39, 50, 51, 62, 65, 89, 90, 91, 119, 136 standard from the library NIST
M18	19.5	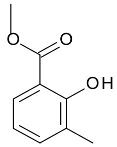	38 (3.0), 50 (6.0), 51 (22.0), 52 (14.0), 77 (23.0), 78 (32.0), 105 (28.0), 106 (100.0), 134 (87.0), 135 (26.0), 166 (37.0)<br>38, 50, 51, 52, 77, 78, 105, 106, 134, 135, 166 standard from the library NIST

M19	18.8	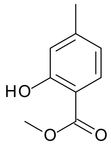	39 (24.0), 51 (20.0), 53 (18.0), 77 (34.0), 78 (34.0), 105 (32.0), 106 (35.0), 134 (100.0),191 (5.0)<br>39, 51, 53, 77, 78, 105, 106, 134, 191 standard from the library NIST
M20	21.6<br>21.6 st	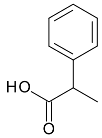	51 (16.0), 63 (9.0), 77 (30.0), 79 (23.0), 91 (23.0), 103 (15.0), 105 (100), 116 (14.0), 150 (30)<br>51 (6.0), 63 (3.0), 77 (15.0), 79 (10.0), 91 (10.0), 103 (11.0), 105 (100), 106 (11.0), 150 (32) measured standard<br>51, 63, 77, 79, 91, 103, 105, 106, 150 standard from the library NIST
M21	22.2	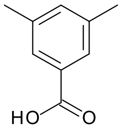	39 (28.0), 51 (21.0), 64 (4.0), 77 (41.0), 78 (13.0), 79 (22.0), 91 (36.0), 103 (16.0), 105 (100.0), 133 (15.0), 150 (52.0)<br>39, 51, 64, 77, 78, 79, 91, 103, 105, 133, 150 standard from the library NIST
M22	23.3	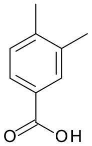	39 (4.0), 51 (24.0), 77 (41.0), 78 (9.0), 79 (18.0), 88 (13.0), 103 (9.0), 104 (7.0), 105 (100.0), 106 (15.0), 133 (28.0), 149 (9.0), 150 (63.0), 151 (4.0)<br>39, 51, 77, 78, 79, 88, 103, 104, 105, 106, 133, 149, 150, 151 standard from the library NIST
M23	24.5	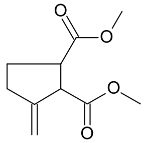	39 (17.0), 41 (15.0), 59 (27.0), 77 (38.0), 78 (24.0), 79 (100.0), 80 (13.0), 106 (8.0), 107 (17.0), 138 (20.0), 166 (60.0), 167 (17.0)<br>39, 41, 59, 77, 78, 79, 80, 106, 107, 138, 166, 167 standard from the library NIST
M24	24.9	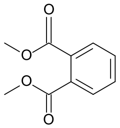	50 (14.0), 76 (16.0), 77 (32.0), 104 (7.0), 133 (7.0), 163 (100.0), 164 (11.0), 194 (6.0)<br>50, 76, 77, 104, 133, 163, 164, 194 standard from the library NIST
M25	25.9<br>25.7 st	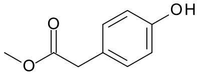	39 (6.0), 51 (8.0), 59 (3.0), 77 (20.0), 107 (100.0), 108 (9.0), 121 (1.0), 134 (1.0), 166 (18.0)<br>39 (5.0), 51 (5.0), 59 (6.0), 77 (23.0), 107 (100.0), 108 (9.0), 121 (2.0), 134 (3.0), 166 (44.0) measured standard<br>39, 51, 59, 77, 107, 108, 121, 134, 166 standard from the library NIST

M26	28.35	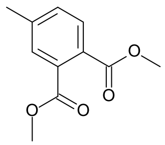	91 (23.0), 149 (8.0), 177 (100.0), 178 (11.0), 206 (0.0)<br>91, 149, 177, 178, 206 standard from the library NIST
M27	32.4	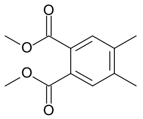	77 (16.0), 105 (11.0), 131.95 (4.0), 163 (3.0), 191 (100.0), 192 (13.0), 222 (16.0)<br>77, 105, 132, 163, 191, 192, 222 standard from the library NIST
M28	30.2	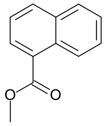	62 (4.0), 74 (9.0), 75 (12.0), 101 (10.0), 126 (26.0), 127 (100.0), 155 (92.0), 156 (12.0), 186 (55.0), 187 (4.0)<br>62, 74, 75, 101, 126, 127, 155, 156, 186, 187 standard from the library NIST
M29	30.3	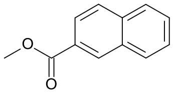	50 (4.0), 51 (21.0), 63 (15.0), 75 (8.0), 77 (15.0), 126 (30.0), 127 (87.0), 128 (18.0), 155 (100.0), 156 (10.0), 186 (78.0), 187 (9.0)<br>50, 51, 63, 75, 77, 126, 127, 128, 155, 156, 186, 187 standard from the library NIST
M30	32.5	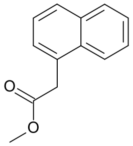	89 (17), 115 (44.0), 139 (10.0), 141 (100.0), 200 (31.0)<br>89, 115, 139, 141, 200 standard from the library NIST
M31	39.01	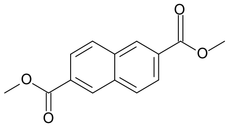	99 (14.0), 126 (33.0), 127 (37.0), 170 (13.0), 185 (10.0), 213 (100.0), 214 (14.0), 244 (40.0), 245 (5.0)<br>99, 126, 127, 170, 185, 213, 214, 244, 245 standard from the library NIST

Supplementary Table S4. Mass spectrometric data of aliphatic and aromatic acids identified during growth on crude oil of *Arthrobacter bussei/agilis* SBUG 2290. These compounds were analysed by GC/MS. The extracted acids were transformed for analytical purposes by methylation to the corresponding methyl esters, some acids occur in unmethylated form**.**

No	t_R (min)	Metabolites	m/z (%) (70 eV) - analyzed with GC/MS
M32	10.8	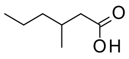	41 (42.0), 42 (22.0), 43 (51.0), 45 (22.0), 55 (28.0), 60 (100.0), 61 (21.0), 69 (9.0), 70 (28.0), 71 (23.0), 73 (16.0), 87 (32.0), 101 (1.0), 115 (3.0), 129 (1.0)<br>41, 42, 43, 45, 55, 60, 61, 69, 70, 71, 73, 101, 115, 129 standard from the library NIST
M33	12.8<br>13.1 st	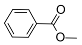	50 (11.0), 51 (30.0), 77 (75.0), 91 (2.0), 92 (3.0), 105 (100.0), 106 (8.0), 136 (31.0)<br>50 (7.0), 51 (17.0), 77 (56.0), 91 (2.0), 92 (2.0), 105 (100.0), 106 (8.0), 136 (38.0) measured standard
M34	14.8<br>15.1 st	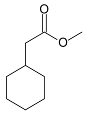	41 (19.0), 43 (25.0), 55 (27.0), 67 (5.0), 74 (100.0), 75 (32.0), 81 (7.0), 97 (4.0), 113 (4.0), 125(6.0), 156 (1.0)<br>41 (9.0), 43 (15.0), 55 (16.0), 67 (7.0), 74 (100.0), 75 (34.0), 81 (6.0), 97 (6.0), 113 (2.0), 125 (9.0), 156 (1.0) measured standard<br>41, 43, 55, 67, 74, 75, 81, 97, 113, 125, 156 standard from the library NIST
M35	16.0	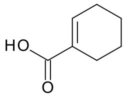	39 (28.0), 41 (44.0), 51 (29.0), 53 (31.0), 67 (11.0), 79 (58.0), 80 (62.0), 81 (100.0), 108 (8.0) 126 (28.0)<br>39, 41, 51, 53, 67, 79, 80, 81, 108, 126 standard from the library NIST
M36	16.3<br>16.5 st	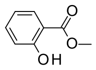	39 (18.0), 63 (27.0), 64 (23.0), 65 (35.0), 92 (87.0), 93 (22.0), 120 (100.0), 121 (29.0), 152 (28.0)<br>39 (12.0), 63 (12.0), 64 (12.0), 65 (19.0), 92 (59.0), 93 (13.0), 120 (100.0), 121 (29.0), 152 (45.0) measured standard<br>39, 63, 64, 65, 92, 93, 120, 121, 152 standard from the library NIST
M37	17.3	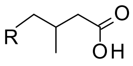	41 (76.0), 42 (22.0), 43 (70.0), 45 (23.0), 55 (31.0), 60 (89.0), 61 (28.0), 69 (23.0), 70 (15.0), 73 (18.0), 87 (85.0), 101 (6.0), 115 (6.0)<br>structure deduced from fragmentation and retention time
M38	19.3	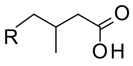	41 (56.0), 42 (19.0), 43 (100.0), 45 (22.0), 55 (36.0), 60 (54.0), 61 (13.0), 69 (36.0), 70 (7.0), 71 (44.0), 73 (19.0), 87 (97.0), 102 (3.0), 113 (12.0)<br>structure deduced from fragmentation and retention time
M39	17.9	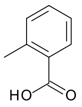	39 (12.0), 41 (18.0), 43 (5.0), 52 (2.0), 59 (5.0), 60 (12.0), 63 (31.0), 65 (56.0), 89 (26.0), 90 (79.0), 91 (100.0), 118 (94.0), 119 (20.0), 136 (75.0)<br>39, 41, 43, 52, 59, 60, 63, 65, 89, 90, 91, 118, 119, 136 standard from the library NIST

M40	18.2	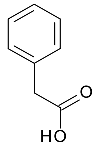	51 (7.0), 62 (6.0), 65 (18.0), 90 (5.0), 91 (100.0), 92 (17.0), 136 (24.0)<br>51, 62, 65, 90, 91, 92, 136 standard from the library NIST
M41	18.3	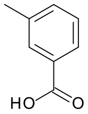	39 (30.0), 50 (8.0), 51 (11.0), 62 (16.0), 63 (16.0), 65 (25.0), 89 (10.0), 90 (6.0), 91(), 92 (16.0), 119 (32.0), 120 (6.0), 135 (5.0), 136 (52.0), 137 (4.0)<br>39, 50, 51, 62, 63, 65, 89, 90, 91,92, 119, 120,135, 136, 137 standard from the library NIST
M42	18.4	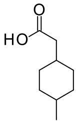	39 (22.0), 40 (3.0), 41 (37.0), 42 (8.0), 54 (9.0), 55 (55.0), 56 (7.0), 60 (36.0), 61 (20.0), 67 (29.0), 68 (12.0), 69 (7.0), 79 (7.0), 81 (50.0), 82 (6.0), 95 (13.0), 96 (25.0), 97 (100.0), 98 (8.0), 156 (1.0)<br>39, 40, 41, 42, 54, 55, 56, 60, 61, 67, 68, 69, 79, 81, 82, 95, 96, 97, 98, 156 standard from the library NIST
M43	18.9	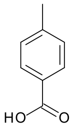	38 (9.0), 41 (22.0), 62 (5.0), 63 (15.0), 64 (6.0), 65 (28.0), 89 (8.0), 90 (7.0), 91 (100.0), 92 (17.0), 119 (52.0), 120 (4.0), 136 (49.0), 137 (6.0)<br>38, 41, 62, 63, 64, 65, 89, 90, 91, 92, 119, 120, 136, 137 standard from the library NIST
M44	19.5	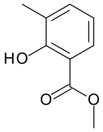	50 (16.0), 51 (24.0), 77 (41.0), 78 (34.0), 79 (12.0), 105 (30.0), 106 (100.0), 107 (22.0), 134 (85.0), 135 (19.0), 166 (42.0)<br>50, 51, 77,78,79,105,106,107,134,135,166 standard from the library NIST
M45	20.2	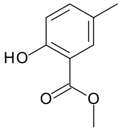	51 (20.0), 52 (7.0), 53 (9.0), 62 (5,0), 77 (39.0), 78 (36.0), 105 (35.0), 106 (39.0), 134 (100.0), 135 (22.0), 151 (2.0), 166 (36.0)<br>51, 52, 53, 62, 77, 78, 105, 106, 134, 135, 151, 166 standard from the library NIST
M46	21.0	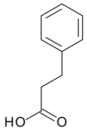	50 (5.0), 62 (7.0), 65 (10.0), 76 (6.0), 78 (17.0), 79 (18.0), 91 (100.0), 92 (16.0), 102 (4.0), 103 (48.0), 104 (21.0), 105 (21.0), 150 (35.0), 151 (3.0)<br>50, 62, 63, 65, 76, 78, 79, 91, 92, 102, 103, 104, 105, 150, 151 standard from the library NIST

M47	21.7	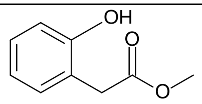	39 (16.0), 40 (3.0), 50 (12.0), 51 (24.0), 52 (13.0), 53 (14.0), 59 (5.0), 61 (5.0), 62 (5.0), 63 (11.0), 77 (74.0), 78 (98.0), 79 (28.0), 105 (9.0), 106 (100.0), 107 (83.0), 108 (6.0), 134 (74.0), 135 (2.0), 165 (2.0), 166 (29.0)<br>39, 40, 50, 51, 52, 53, 59, 61, 62, 63, 77, 78, 79, 105, 106, 107, 108, 134, 135, 165, 166 standard from the library NIST
M48	21.9	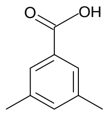	39 (13.0), 40 (4.0), 50 (9.0), 51 (25.0), 52 (5.0), 53 (11.0), 62 (7.0), 63 (17.0), 64 (4.0), 65 (11.0), 77 (42.0), 78 (11.0), 79 (15.0), 89 (6.0), 91 (42.0), 103 (19.0), 104 (9.0), 105 (100.0), 106 (10.0), 107 (8.0), 132 (6.0), 133 (30.0), 134 (2.0), 135 (11.0), 150 (56.0)<br>39, 40, 50, 51, 52, 53, 62, 63, 64, 65, 77, 78, 79, 89, 91, 103, 104, 105, 106, 107, 132, 133, 134, 135, 150 standard from the library NIST
M49	23.0	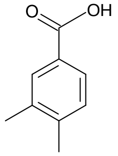	50 (3.0), 51 (20.0), 77 (36.0), 78 (9.0), 79 (7.0), 91 (38.0), 103 (14.0), 104 (8.0), 105 (100.0), 106 (14.0), 107 (4.0), 132 (4.0), 133 (22.0), 134 (3.0), 149 (7.0), 150 (62.0)<br>50, 51, 77, 78, 79, 91, 103, 104, 105, 106, 107, 132, 133, 134, 149, 150 standard from the library NIST
M50	23.2	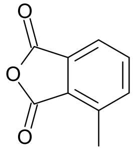	39 (6.0), 50 (19.0), 63 (45.0), 64 (10.0), 78 (8.0), 89 (75.0), 90 (100.0), 105 (1.0), 106 (13.0), 118 (71.0), 134 (39.0), 135 (10.0), 162 (56.0), 163 (7.0)<br>39, 50, 63, 64, 78, 89, 90, 105, 106, 118, 134, 135, 162, 163 standard from the library NIST
M51	24.1	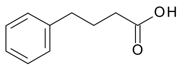	39 (13.0), 41 (6.0), 45 (7.0), 52 (2.0), 55 (12.0), 58 (6.0), 60 (12.0), 62 (4.0), 63 (8.0), 64 (2.0), 65 (28.0), 77 (4.0), 78 (12.0), 79 (9.0), 89 (6.0), 91 (71.0), 92 (6.0), 104 (100.0), 105 (26.0), 146 (9.0), 164(10.0)<br>39, 41, 45, 52, 55, 58, 60, 62, 63, 64, 65, 77, 78, 79, 89, 91, 92, 104, 105, 146, 164 standard from the library NIST

Supplementary Table S5. Mass spectrometric data of aliphatic and aromatic acids identified during growth on crude oil of *Kocuria polaris* SBUG 2288. These compounds were analysed by GC/MS. The extracted acids were transformed for analytical purposes by methylation to the corresponding methyl esters, some acids occur in unmethylated form.

No	t_R (min)	Metabolites	m/z (%) (70 eV) - analyzed with GC/MS
M52	11.5	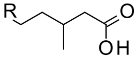	41 (17.0), 42 (14.0), 45 (21.0), 59 (9.0), 60 (100.0), 61 (20.0), 69 (17.0), 70 (23.0), 73 (20.0), 87 (41.0)<br>41, 42, 45, 59, 60, 61, 69, 70, 73, 87 standard from the library NIST
M53	11.6<br>11.9 st	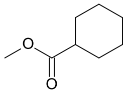	41 (43.0), 55 (100.0), 67 (24.0), 68 (18.0), 69 (27.0), 74 (33.0), 81 (24.0), 82 (23.0), 83 (78.0), 87 (77.0), 101 (11.0), 110 (27.0), 111 (32.0), 113 (23.0), 127 (4.0), 142 (24.0)<br>41 (32.0), 55 (92.0), 67 (21.0), 68 (19.0), 69 (13.0), 74 (39.0), 81 (17.0), 82 (28.0), 83 (81.0), 87 (100.0), 101 (17.0), 110 (31.0), 111 (25.0), 113 (26.0), 127 (10.0), 142 (40.0) measured standard<br>41, 55, 67, 68, 69, 74, 81, 82, 83, 87, 101, 110, 111, 113, 127, 142 standard from the library NIST
M54	12.8<br>13.1 st	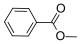	50 (17.0), 51 (29.0), 77 (71.0), 91 (1.0), 92 (2.0), 105 (100.0), 106 (10.0), 136 (34.0)<br>50 (7.0), 51 (17.0), 77 (56.0), 91 (2.0), 92 (2.0), 105 (100.0), 106 (8.0), 136 (38.0) measured standard<br>50, 51, 63, 77, 92, 105, 106, 136 standard from the library NIST
M55	14.9	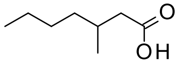	39 (17.0), 41 (45.0), 42 (18.0), 43 (56.0), 45 (24.0), 56 (15.0), 59 (10.0), 60 (100.0), 61 (23.0), 71 (1.0), 84 (36.0), 85 (34.0), 87 (68.0), 101 (5.0), 125 (2.0) standard from the library NIST
M56	16.6	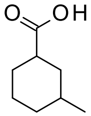	39 (52.0), 41 (70.0), 42 (17.0), 43 (44.0), 45 (22.0), 53 (22.0), 54 (15.0), 55 (100.0), 56 (23.0), 67 (28.0), 69 (27.0), 70 (49.0), 73 (29.0), 82 (43.0), 83 (21.0), 97 (25.0), 99 (13.0), 113 (23.0), 142 (17.0)<br>39, 41, 42, 43, 45, 53, 54, 55, 56, 67, 69, 70, 73, 82, 83, 97, 99, 113, 142 standard from the library NIST
M57	17.4<br>17.6 st	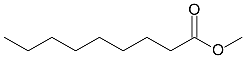 C9	39 (3.0), 41 (17.0), 55 (18.0), 59 (15.0), 71 (8.0), 73 (3.0), 74 (100.0), 75 (4.0), 83 (6.0), 87 (51.0), 88 (5.0), 101 (7.0), 129 (8.0), 141 (11.0), 142 (9.0), 172 (1.0)<br>39 (5.0), 41 (15.0), 55 (17.0), 59 (11.0), 71 (4.0), 73 (2.0), 74 (100.0), 75 (8.0), 83 (3.0), 87 (48.0), 88 (4.0), 101 (6.0), 129 (11.0), 141 (11.0), 143 (9.0), 172 (1.0) measured standard<br>39, 41, 55, 59, 71, 73, 74, 75, 83, 87, 88, 101, 129, 141, 142, 172 standard from the library NIST
M58	17.7	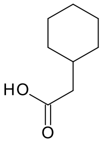	39 (41.0), 41 (53.0), 42 (13.0), 43 (19.0), 45 (1.0), 54 (23.0), 55 (79.0), 60 (100.0), 61 (60.0), 67 (57.0), 81 (16.0), 82 (67.0), 83 (61.0), 142 (3.0)<br>39, 41, 42, 43, 45, 54, 55, 60, 61, 67, 81, 82, 83, 142 standard from the library NIST

M59	18.7	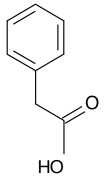	51 (6.0), 63 (11.0), 65 (20.0), 91 (100.0), 92 (14.0), 136 (24.0)<br>51, 63, 65, 91, 92, 136 standard from the library NIST
M60	19.1	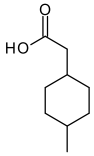	39 (20.0), 41 (36.0), 42 (10.0), 43 (15.0), 45 (9.0), 53 (11.0), 54 (12.0), 55 (63.0), 60 (39.0), 61 (19.0), 67 (17.0), 68 (11.0), 69 (15.0), 79 (9.0), 81 (43.0), 95 (13.0), 96 (21.0), 97 (100.0), 98 (8.0), 138 (2.0) 156 (1.0)<br>39, 41, 42, 43, 45, 53, 54, 55, 60, 61, 67, 68, 69, 79, 81, 95, 96, 97, 98, 138, 156 standard from the library NIST
M61	19.5	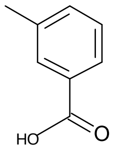	39 (11.0), 63 (15.0), 65 (22.0), 89 (11.0), 90 (9.0), 91 (100.0), 92 (17.0), 136 (54.0)<br>39, 63, 65, 89, 90, 91, 92, 136 standard from the library NIST
M62	20.3	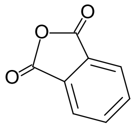	37 (10.0), 38 (11.0), 50 (46.0), 74 (15.0), 75 (12.0), 76 (92.0), 104 (100.0), 148 (17.0)<br>37, 38, 50, 74, 75, 76, 104, 148 standard from the library NIST
M63	20.7<br>20.8 st	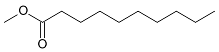 C10	39 (3.0), 69 (24.0), 74 (100.0), 75 (11.0), 87 (56.0), 97 (17.0), 101 (9.0), 143 (14.0), 186 (2.0)<br>39 (4.0), 69 (8.0), 74 (100.0), 75 (10.0), 87 (53.0), 97 (3.0), 101 (7.0), 143 (18.0), 186 (2.0) measured standard<br>39, 69, 74, 75, 87, 97, 101, 143, 186 standard from the library NIST
M64	21.4	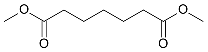	39 (23.0), 40 (5.0), 41 (42.0), 42 (31.0), 43 (46.0), 44 (4.0), 45 (13.0), 48 (7.0), 49 (6.0), 53 (6.0), 55 (91.0), 56 (4.0), 57 (5.0), 59 (79.0), 67 (4.0), 68 (17.0), 69 (59.0), 73 (39.0), 74 (89.0), 83 (44.0), 85 (8.0), 87 (29.0), 97 (30.0), 100 (16.0), 111 (17.0), 115 (100.0), 156 (8.0), 157 (38.0)<br>39, 40, 41, 42, 43, 44, 45, 48, 49, 53, 55, 56, 57, 59, 67, 68, 69, 73, 74, 83, 85, 87, 97, 100, 111, 115, 156, 157 standard from the library NIST
M65	21.5	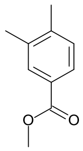	77 (23.0), 78 (7.0), 103 (17.0), 105 (38.0), 133 (100.0), 134 (6.0), 164 (29.0)<br>77, 78, 103, 105, 133, 134, 164 standard from the library NIST

M66	21.7<br>21.6 st	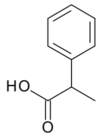	39 (6.0), 51 (9.0), 63 (5.0), 77 (22.0), 79 (14.0), 91 (14.0), 103 (12.0), 105 (100.0), 106 (11.0), 150 (29.0)<br>39 (4.0), 51 (6.0), 63 (3.0), 77 (15.0), 79 (10.0), 91 (10.0), 103 (11.0), 105 (100), 106 (11.0), 150 (32) measured standard<br>39, 51, 63, 77, 79, 91, 103, 105, 106, 150 standard from the library NIST
M67	22.3	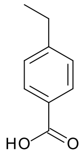	50 (9.0), 51 (17.0), 77 (34.0), 78 (8.0), 79 (6.0), 89 (9.0), 91 (24.0), 103 (16.0), 104 (10.0), 105 (100.0), 106 (11.0), 107 (20.0), 135 (36.0), 150 (62.0)<br>50, 51, 77, 78, 79, 89, 91, 103, 104, 105, 106, 107, 135, 150 standard from the library NIST
M68	22.6<br>22.4 st	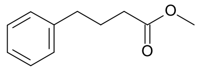	39 (7.0), 43 (6.0), 51 (8.0), 65 (21.0), 74 (60.0), 77 (8.0), 78 (8.0), 89 (17.0), 91 (59.0), 92 (6.0), 103 (8.0), 104 (100.0), 105 (31.0), 115 (6.0), 117 (7.0), 146 (17.0), 147 (15.0), 178 (18.0)<br>39 (10.0), 43 (24.0), 51 (9.0), 65 (27.0), 74 (96.0), 77 (11.0), 78 (10.0), 89 (17.0), 91 (100.0), 92 (9.0), 103 (9.0), 104 (100.0), 105 (43.0), 115 (10.0), 117 (10.0), 146 (36.0), 147 (34.0), 178 (30.0) measured standard<br>39, 43, 51, 65, 74, 77, 78, 89, 91, 92, 103, 104, 105, 115, 117, 146, 147, 178 standard from the library NIST
M69	24.6	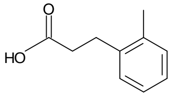	39 (14.0), 65 (13.0), 77 (29.0), 78 (10.0), 79 (21.0), 89 (7.0), 104 (28.0), 105 (100.0), 106 (9.0), 107 (5.0), 117 (14.0), 118 (17.0), 119 (9.0), 164 (39.0)<br>39, 65, 77, 78, 79, 90, 104, 105, 106, 107, 117, 118, 119, 164 standard from the library NIST
M70	24.9	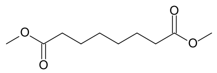	41 (66.0), 42 (21.0), 43 (52.0), 45 (11.0), 55 (100.0), 56 (18.0), 57 (18.0), 59 (71.0), 67 (12.0), 68 (20.0), 69 (94.0), 74 (89.0), 82 (16.0), 83 (49.0), 84 (12.0), 87 (40.0), 97 (74.0), 110 (20.0), 129 (77.0), 138 (72.0), 139 (18.0), 142 (10.0), 171 (46.0)<br>41, 42, 43, 45, 55, 56, 57, 59, 67, 68, 69, 74, 82, 83, 84, 87, 97, 110, 129, 138, 139, 142, 171 standard from the library NIST
M71	27.8	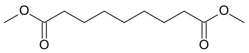	41 (49.0), 42 (21.0), 43 (37.0), 55 (100.0), 56 (10.0), 57 (9.0), 59 (59.0), 67 (13.0), 68 (10.0), 69 (39.0), 73 (8.0), 74 (75.0), 81 (12.0), 82 (12.0), 83 (66.0), 84 (27.0), 96 (13.0), 97 (30.0), 111 (50.0), 124 (25.0), 125 (19.0), 143 (34.0), 152 (77.0), 153 (10.0), 185 (39.0)<br>41, 42, 43, 55, 56, 57, 59, 67, 68, 69,73, 74, 81, 82, 83, 84, 96, 97, 111, 124, 125, 143, 152, 153, 185 standard from the library NIST
M72	30.22	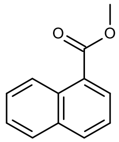	63 (12.0), 74 (28.0), 75 (13.0), 76 (10.0), 77 (23.0), 126 (28.0), 127 (100.0), 128 (14.0), 155 (97.0), 186 (68.0)<br>63, 74, 75, 76, 77, 126, 127, 128, 155, 186 standard from the library NIST

M73	30.23	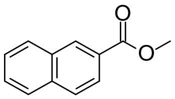	51 (15.0), 63 (11.0), 74 (18.0), 75 (18.0), 76 (10.0), 77 (30.0), 126 (20.0), 127 (91.0), 128 (16.0), 155 (100.0), 156 (14.0), 185 (5.0), 186 (72.0)<br>51, 63, 74, 75, 76, 77, 126, 127, 128, 155, 156, 185, 186 standard from the library NIST
M74	30.6	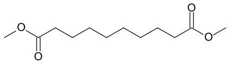	39 (15.0), 41 (55.0), 42 (16.0), 43 (57.0), 53 (6.0), 54 (5.0), 55 (100.0), 56 (19.0), 57 (62.0), 59 (46.0), 68 (7.0), 70 (12.0), 74 (69.0), 75 (4.0), 81 (11.0), 84 (36.0), 87 (27.0), 96 (12.0), 97 (46,0), 98 (55.0), 99 (11.0), 125 (46.0), 138 (28.0), 139 (9.0), 157 (22.0), 167 (2.0), 199 (29.0)<br>39, 41, 42, 43, 53, 54, 55, 56, 57, 59, 68, 70, 74, 75, 81, 84, 87, 96, 97, 98, 99, 125, 138, 139, 157, 167, 199 standard from the library NIST
M75	32.6<br>32.4 st	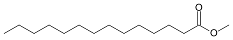<br>C14	41 (26.0), 43 (30.0), 56 (12.0), 59 (11.0), 69 (30.0), 74 (100.0), 75 (14.0), 87 (60.0), 143 (12.0), 199 (5.0), 242 (5.0)<br>41 (21.0), 43 (25.0), 57 (11.0), 59 (9.0), 69 (12.0), 74 (100.0), 75 (16.0), 87 (65.0), 143 (17.0), 199 (11.0), 242 (6.0) measured standard<br>41, 43, 56, 59, 69, 74, 75, 87, 143, 199, 242 standard from the library NIST
M76	34.5	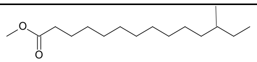	41 (9.0), 42 (2.0), 43 (15.0), 55 (24.0), 75 (23.0), 95 (4.0), 143 (15.0), 199 (2.0)<br>41, 42, 43, 55, 75, 95, 143, 199 standard from the library NIST
M77	48.7	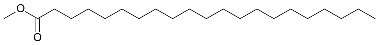<br>C21	43 (51.0), 55 (15.0), 57 (37.0), 70 (2.0), 74 (100.0), 87 (87.0), 143 (24.0), 297 (10.0), 340 (14.0)<br>43, 55, 57, 70, 74, 87, 143, 297, 340 standard from the library NIST
M78	50.7	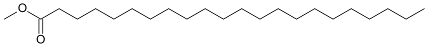<br>C22	41 (31.0), 42 (5.0), 43 (41.0), 56 (8.0), 58 (4.0), 69 (21.0), 71 (13.0), 74 (100.0), 75 (27.0), 83 (14.0), 111 (8.0), 143 (19.0), 255 (4.0), 311 (8.0), 354 (12.0)<br>41, 42, 43, 56, 58, 69, 71, 74, 75, 83, 111, 143, 255, 311, 354 standard from the library NIST
M79	52.5	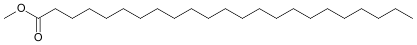<br>C23	41 (24.0), 42 (7.0), 43 (100.0), 55 (26.0), 56 (12.0), 57 (81.0), 69 (31.0), 71 (50.0), 74 (89.0), 75 (29.0), 87 (71.0), 143 (19.0), 325 (6.0), 368 (11.0)<br>41, 42, 43, 55, 56, 57, 69, 71, 74, 75, 87, 143, 325, 368 standard from the library NIST

Supplementary Table S6. Mass spectrometric data of aliphatic and aromatic acids identified during growth on crude oil of *Kocuria rosea* SBUG 2287. These compounds were analysed by GC/MS. The extracted acids were transformed for analytical purposes by methylation to the corresponding methyl esters, some acids occur in unmethylated form.

No	t_R (min)	Metabolites	m/z (%) (70 eV) - analyzed with GC/MS
M80	8.7<br>9.1 st	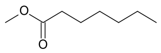	43 (29.0), 74 (100.0), 87 (17.0), 101 (21.0), 113 (10.0), 144 (1.0)<br>43 (27.0), 74 (100.0), 87 (36.0), 101 (15.0), 113 (16.0), 144 (1.0) measured standard<br>43, 74, 87, 101, 113, 144 standard from the library NIST
M81	11.6<br>11.9 st	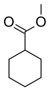	55 (60.0), 67 (23.0), 68 (12.0), 74 (38.0), 81 (17.0), 82 (27.0), 83 (66.0), 87 (100.0), 101 (15.0), 110 (36.0), 111 (22.0), 127 (8.0), 142 (32.0)<br>55 (92.0), 67 (21.0), 68 (19.0), 74 (39.0), 81 (17.0), 82 (28.0), 83 (81.0), 87 (100.0), 101 (17.0), 110 (31.0), 111 (25.0), 127 (10.0), 142 (40.0) measured standard<br>55, 67, 68, 74, 81, 82, 83, 87, 101, 110, 111, 127, 142 standard from the library NIST
M82	12.8<br>13.1 st	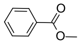	50 (16.0), 51 (33.0), 77 (77.0), 91 (3.0), 92 (3.0), 105 (100.0), 106 (7.0), 136 (29.0)<br>50 (7.0), 51 (17.0), 77 (56.0), 91 (2.0), 92 (2.0), 105 (100.0), 106 (8.0), 136 (38.0) measured standard<br>50, 51, 77, 91, 92, 105, 106, 136 standard from the library NIST
M83	14.8<br>15.1 st	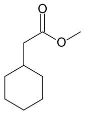	41 (15.0), 43 (22.0), 55 (20.0), 67 (8.0), 74 (100.0), 75 (34.0), 81 (6.0), 97 (5.0), 113 (2.0), 125 (6.0), 156 (1.0)<br>41 (9.0), 43 (15.0), 55 (16.0), 67 (7.0), 74 (100.0), 75 (34.0), 81 (6.0), 97 (6.0), 113 (2.0), 125 (9.0), 156 (1.0) measured standard<br>41, 43, 55, 67, 74, 75, 81, 97, 113, 125, 156 standard from the library NIST
M84	16.6<br>17.1 st	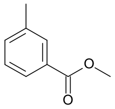	39 (10.0), 50 (4.0), 51 (6.0), 63 (11.0), 65 (23.0), 77 (2.0), 89 (10.0), 90 (5.0), 91 (62.0), 105 (1.0), 119 (100.0), 120 (8.0), 135 (1.0), 150 (29.0)<br>39 (4.0), 50 (2.0), 51 (3.0), 63 (6.0), 65 (15.0), 77 (1.0), 89 (7.0), 90 (4.0), 91 (54.0), 105 (2.0), 119 (100.0), 120 (9.0), 135 (2.0), 150 (40.0) measured standard<br>39, 50, 51, 63, 65, 77, 89, 90, 91, 105, 119, 120, 135, 150 standard from the library NIST
M85	16.9	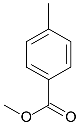	39 (11.0), 63 (9.0), 65 (21.0), 89 (10.0), 91 (58.0), 119 (100.0), 120 (13.0), 150 (35.0)<br>39, 63, 65, 89, 91, 119, 120, 150 standard from the library NIST
M86	17.5	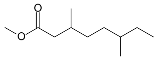	41 (30.0), 43 (36.0), 55 (24.0), 56 (6.0), 57 (14.0), 69 (23.0), 74 (100.0), 75 (15.0), 83 (4.0), 101 (61.0), 129 (3.0), 155, (3.0), 171 (2.0), 186 (1.0)<br>41, 43, 55, 56, 57, 69, 74, 75, 83, 101, 129, 155, 171 186 standard from the library NIST

M87	20.3	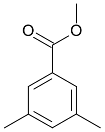	77 (24.0), 78 (8.0), 79 (16.0), 103 (12.0), 104 (4.0), 105 (46.0), 133 (100.0), 134 (14.0), 164 (34.0)<br>77, 78, 79, 103, 104, 105, 133, 134, 164 standard from the library NIST
M88	20.7<br>20.8 st	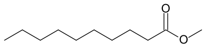 C10	59 (19.0), 74 (100.0), 75 (14.0), 87 (55.0), 101 (12.0), 143 (9.0), 186 (2.0)<br>59 (10.0), 74 (100.0), 75 (10.0), 87 (53.0), 101 (7.0), 143 (18.0), 186 (2.0) measured standard<br>59, 74, 75, 87, 101, 143, 186 standard from the library NIST
M89	21.5	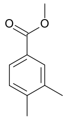	51 (8.0), 63 (5.0), 77 (17.0), 78 (8.0), 79 (14.0), 91 (4.0), 103 (11.0), 105 (30.0), 119 (5.0), 133 (100.0), 134 (10.0), 148 (1.0), 164 (28.0)<br>51, 63, 77, 78, 79, 91, 103, 105, 119, 133, 134, 148, 164 standard from the library NIST
M90	24.8	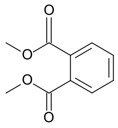	50 (11.0), 76 (14.0), 77 (19.0), 92 (14.0), 135 (10.0), 163 (100.0), 164 (12.0), 194 (6.0)<br>50, 76, 77, 92, 135, 163, 164, 194 standard from the library NIST
M91	27.0<br>27.5st	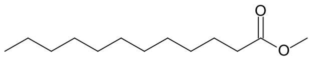 C12	41 (35.0), 43 (36.0), 55 (33.0), 74 (100.0), 75 (12.0), 87 (60.0), 143 (9.0), 171 (8.0), 214 (3.0)<br>41 (19.0), 43 (20.0), 55 (21.0), 74 (100.0), 75 (12.0), 87 (65.0), 143 (16.0), 171 (13.0), 214 (5.0) measured standard<br>41, 43, 55, 74, 75, 87, 143, 171, 214 standard from the library NIST
M92	30.2	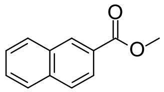	74 (8.0), 75 (11.0), 77 (17.0), 126 (21.0), 127 (100.0), 128 (8.0), 155 (90.0), 156 (13.0), 186 (48.0), 187 (7.0)<br>74, 75, 77, 126, 127, 128, 155, 156, 186, 187 standard from the library NIST
M93	32.6<br>32.4st	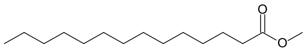 C14	41 (28.0), 43 (33.0), 55 (29.0), 57 (19.0), 59 (10.0), 69 (15.0), 74 (100.0), 75 (14.0), 87 (60.0), 143 (14.0), 199 (9.0), 242 (2.0)<br>41 (21.0), 43 (25.0), 55 (23.0), 57 (11.0), 59 (9.0), 69 (12.0), 74 (100.0), 75 (16.0), 87 (65.0), 143 (17.0), 199 (11.0), 242 (6.0) measured standard<br>41, 43, 55, 57, 59, 69, 74, 75, 87, 143, 199, 242 standard from the library NIST
M94	34.5<br>34.3 st	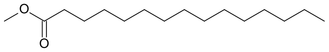 C15	74 (100.0), 75 (17.0), 87 (64.0), 143 (11.0), 213 (7.0), 256 (4.0)<br>74 (100.0), 75 (17.0), 87 (71.0), 143 (20.0), 213 (14.0), 256 (11.0) measured standard<br>74, 75, 87, 143, 213, 256 standard from the library NIST

Supplementary Table S7. Mass spectrometric data of aliphatic and aromatic acids identified during growth on crude oil of *Bacillus subtilis* SBUG 2285. These compounds were analysed by GC/MS. The extracted acids were transformed for analytical purposes by methylation to the corresponding methyl esters, some acids occur in unmethylated form.

No	t_R (min)	Metabolites	m/z (%) (70 eV) - analyzed with GC/MS
M95	4.7	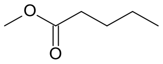	41 (28.0), 42 (8.0), 43 (28.0), 55 (16.0), 56 (7.0), 57 (36.0), 58 (5,0), 59 (24.0), 74 (100.0), 86 (2.0), 101 (2.0), 116 (1.0)<br>41, 42, 43, 55, 56, 57, 58, 59, 74, 86, 101, 116 standard from the library NIST
M96	6.3	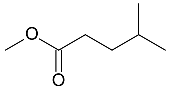	39 (15.0), 41 (53.0), 42 (10.0), 43 (80.0), 55 (70.0), 56 (15.0), 57 (50.0), 59 (28.0), 69 (13.0), 70 (8.0), 71 (5.0), 73 (34.0), 74 (100.0), 81 (36.0), 83 (13.0), 87 (60.0), 88 (19.0), 99 (26.0), 101 (9.0), 115 (4.0), 130 (1.01)<br>39, 41, 42, 43, 55, 56, 57, 59, 69, 70, 71, 73, 74, 81, 83, 87, 88, 99, 101, 115, 130 standard from the library NIST
M97	7.2	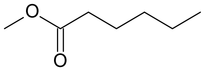	39 (22.0), 41 (42.0), 42 (18.0), 43 (63.0), 55 (36.0), 57 (31.0), 59 (26.0), 74 (100.0), 87 (36.0), 99 (22.0), 101 (10.0), 130 (1.0)<br>39, 41, 42, 43, 55, 57, 59, 74, 87, 99, 101, 130 standard from the library NIST
M98	8.5	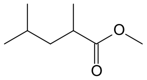	39 (17.0), 41 (25.0), 43 (27.0), 55 (26.0), 56 (10.0), 57 (15.0), 59 (15.0), 88 (100.0), 101 (20.0), 144 (1.0)<br>39, 41, 43, 55, 56, 57, 59, 88, 101, 144 standard from the library NIST
M99	8.7	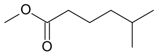	41 (20.0), 42 (9.0), 43 (43.0), 59 (17.0), 74 (100.0), 75 (10.0) 101 (15.0), 102 (2.0), 144 (1.0)<br>41, 42, 43, 59, 74, 75, 101, 102, 144 standard from the library NIST
M100	10.1	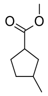	39 (13.0), 41 (14.0), 55 (49.0), 67 (20.0), 83 (24.0), 87 (100.0), 100 (22.0), 111 (6.0), 127 (7.0), 142 (6.0)<br>39, 41, 55, 67, 83, 87, 100, 111, 127, 142 standard from the library NIST
M101	11.1	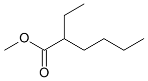	41 (35.0), 42 (9.0), 57 (28.0), 58 (4.0), 69 (49.0), 87 (100.0), 101 (16.0), 102 (93.0), 115 (13.0), 130 (16.0)<br>41, 42, 57, 58, 69, 87, 101, 102, 115, 130 standard from the library NIST
M102	11.6	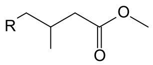	39 (14.0), 41 (28.0), 42 (9.0), 43 (41.0), 55 (17.0), 56 (5.0), 57 (9.0), 59 (26.0), 69 (12.0), 71 (10.0), 73 (7.0), 74 (100.0), 75 (7.0), 99 (15.0), 101 (11.0)<br>39, 41, 42, 43, 55, 56, 57, 69, 71, 73, 74, 75, 99, 101 structure deduced from fragmentation and retention time
M103	11.6<br>11.9 st	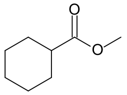	41 (7.0), 55 (100.0), 67 (29.0), 68 (23.0), 69 (5.0), 74 (41.0), 81 (21.0), 82 (31.0), 83 (82.0), 87 (95.0), 101 (13.0), 110 (26.0), 111 (24.0), 113 (10.0), 142 (31.0)<br>41 (32.0), 55 (92.0), 67 (21.0), 68 (19.0), 69 (13.0), 74 (39.0), 81 (17.0), 82 (28.0), 83 (81.0), 87 (100.0), 101 (17.0), 110 (31.0), 111 (25.0), 113 (26.0), 142 (40.0) measured standard<br>41, 55, 67, 68, 69, 74, 81, 82, 83, 87, 101, 110, 111, 113, 142 standard from the library NIST

M104	11.8	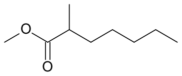	41 (32.0), 42 (13.0), 43 (50.0), 55 (13.0), 56 (15.0), 57 (74.0), 88 (100.0), 101 (18.0), 115 (6.0), 143 (1.0), 158 (1.0)<br>41, 42, 43, 55, 56, 57, 88, 101, 115, 143, 158 standard from the library NIST
M105	12.8<br>13.1 st	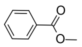	50 (17.0), 51 (29.0), 77 (71.0), 91 (3.0), 92 (2.0), 105 (100.0), 106 (10.0), 136 (34.0)<br>50 (7.0), 51 (17.0), 77 (56.0), 91 (2.0), 92 (2.0), 105 (100.0), 106 (8.0), 136 (38.0) measured standard<br>50, 51, 77, 91, 92, 105, 106, 136 standard from the library NIST
M106	13.83	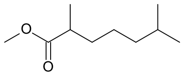	41 (18.0), 42 (8.0), 43 (17.0), 55 (21.0), 56 (8.0), 57 (18.0), 71 (8.0), 87 (14.0), 88 (100.0), 101 (31.0), 129 (4.0), 141 (3.0), 157 (2.0), 172 (1.0)<br>41, 42, 43, 55, 56, 57, 71, 87, 88, 101, 129, 141, 157, 172 standard from the library NIST
M107	13.9	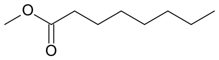	39 (7.0), 59 (17.0), 74 (100.0), 87 (38.0), 115 (9.0), 127 (5.0), 158 (1.0)<br>39, 59, 74, 87, 115, 127, 158 standard from the library NIST
M108	14.8<br>15.1 st	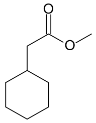	41 (15.0), 43 (22.0), 55 (20.0), 67 (8.0), 74 (100.0), 75 (34.0), 81 (5.0), 97 (5.0), 113 (2.0), 125 (6.0), 156 (1.0)<br>41 (9.0), 43 (15.0), 55 (16.0), 67 (7.0), 74 (100.0), 75 (34.0), 81 (6.0), 97 (6.0), 113 (2.0), 125 (9.0), 156 (1.0) measured standard<br>41, 43, 55, 67, 74, 75, 81, 97, 113, 125, 156 standard from the library NIST
M109	15.2	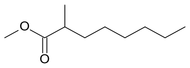	41 (16.0), 55 (20.0), 88 (100.0), 101 (21.0), 115 (6.0), 129 (3.0), 143 (2.0), 157 (1.0), 172 (1.0)<br>41, 55, 88, 101, 115, 129, 143, 157, 172 standard from the library NIST
M110	15.4	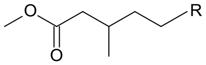	41 (32.0), 43 (32.0), 55 (26), 56 (10.0), 57 (18.0), 59 (15), 67 (7.0), 69 (19.0), 74 (100.0), 75 (15.0), 101 (31.0), 123 (2.0)<br>41, 43, 55, 56, 57, 59, 67, 69, 74, 75, 101, 123 structure deduced from fragmentation and retention time
M111	15.7<br>16.1 st	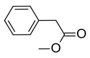	39 (8.0), 51 (4.0), 59 (6.0), 65 (16.0), 77 (1.0), 89 (6.0), 91 (100.0), 92 (7.0), 105 (1.0), 119 (5.0), 150 (23.0)<br>39 (4.0), 51 (2.0), 59 (5.0), 65 (12.0), 77 (1.0), 89 (5.0), 91 (100.0), 92 (8.0), 105 (1.0), 119 (2.0), 150 (34.0) measured standard<br>39, 51, 59, 65, 77, 89, 91, 92, 105, 119, 150 standard from the library NIST
M112	16.3<br>16.5 st	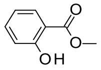	39 (19.0), 63 (22.0), 64 (23.0), 65 (25.0), 92 (75.0), 93 (20.0), 120 (100.0), 121 (32.0), 152 (28.0)<br>39 (12.0), 63 (12.0), 64 (12.0), 65 (19.0), 92 (59.0), 93 (13.0), 120 (100.0), 121 (29.0), 152 (45.0) measured standard<br>39, 63, 64, 65, 92, 93, 120, 121, 152 standard from the library NIST
M113	16.6<br>17.1 st	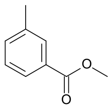	39 (10.0), 50 (3.0), 51 (6.0), 63 (11.0), 65 (24.0), 77 (2.0), 89 (10.0), 90 (5.0), 91 (64.0), 105 (2.0), 119 (100.0), 120 (9.0), 135 (1.0), 150 (29.0)<br>39 (4.0), 50 (2.0), 51 (3.0), 63 (6.0), 65 (15.0), 77 (1.0), 89 (7.0), 90 (4.0), 91 (54.0), 105 (2.0), 119 (100.0), 120 (9.0), 135 (2.0), 150 (40.0) measured standard<br>39, 50, 51, 63, 65, 77, 89, 90, 91, 105, 119, 120, 135, 150 standard from the library NIST

M114	16.9	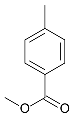	39 (11.0), 63 (9.0), 65 (21.0), 89 (10.0), 91 (58.0), 119 (100.0), 120 (13.0), 150 (35.0)<br>39, 63, 65, 89, 91, 119, 120, 150 standard from the library NIST
M115	17.5	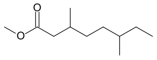	41 (36.0), 43 (35.0), 55 (26.0), 56 (8.0), 57 (15.0), 69 (28.0), 74 (100.0), 75 (15.0), 83 (7.0), 101 (68.0), 129 (3.0), 155, (5.0), 171 (1.0), 186 (1.0)<br>41, 43, 55, 56, 57, 69, 74, 75, 83, 101, 129, 155, 171 186 standard from the library NIST
M116	19.1	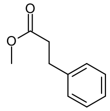	39 (5.0), (51 (9.0), 77 (24.0), 78 (12.0), 79 (13.0), 91 (57.0), 103 (15.0), 104 (100.0), 105 (59.0), 133 (7.0), 164 (25.0)<br>39, 51, 77, 78, 79, 91, 103, 104, 105, 133, 164 standard from the library NIST
M117	19.8	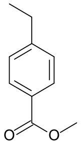	51 (9.0), (77 (33.0), 79 (20.0), 103 (18.0), 105 (78.0), 133 (100.0), 149 (18.0), 164 (54.0)<br>51, 77, 79, 103, 105, 133, 149, 164 standard from the library NIST
M118	20.3	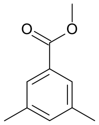	77 (24.0), 78 (8.0), 79 (16.0), 103 (12.0), 104 (4.0), 105 (46.0), 133 (100.0), 134 (14.0), 164 (34.0)<br>77, 78, 79, 103, 104, 105, 133, 134, 164 standard from the library NIST
M119	20.7<br>20.8 st	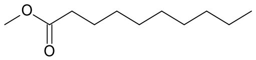 C10	59 (14.0), 74 (100.0), 75 (13.0), 87 (51.0), 97 (9.0), 101 (4.0), 143 (12.0), 186 (2.0)<br>59 (10.0), 74 (100.0), 75 (10.0), 87 (53.0), 97 (3.0), 101 (7.0), 143 (18.0), 186 (2.0) measured standard<br>59, 75, 87, 97, 101, 143, 186 standard from the library NIST<br>59, 74, 75, 87, 97, 101, 143, 186 standard from the library NIST
M120	21.5	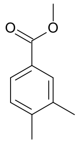	77 (21.0), 78 (7.0), 79 (11.0), 103 (12.0), 104 (6.0), 105 (40.0), 133 (100.0), 134 (9.0), 164 (33.0)<br>77, 78, 79, 103, 104, 105, 133, 134, 164 standard from the library NIST
M121	22.3	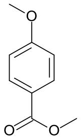	63 (14.0), 64 (13.0), 77 (29.0), 92 (20.0), 107 (13.0), 135 (100.0), 166 (32.0)<br>63, 64, 77, 92, 107, 135, 166 standard from the library NIST

M122	24.8	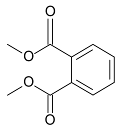	50 (13.0), 76 (14.0), 77 (24.0), 92 (17.0), 121 (22.0), 135 (5.0), 163 (100.0), 164 (8.0), 194 (6.0)<br>50, 76, 77, 92, 121 135, 163, 164, 194 standard from the library NIST
M123	26.97	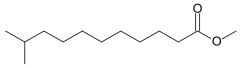	69 (20.0), 74 (100.0), 75 (12.0), 87 (60.0), 143 (8.0), 171 (8.0), 214 (2.0)<br>69, 74, 75, 87, 143, 171, 214 standard from the library NIST
M124	27.7	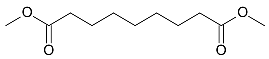	41 (52.0), 42 (22.0), 43 (44.0), 55 (100.0), 56 (11.0), 57 (9.0), 59 (56.0), 67 (11.0), 68 (11.0), 69 (42.0), 73 (10.0), 74 (75.0), 81 (8.0), 82 (18.0), 83 (64.0), 84 (26.0), 96 (13.0), 97 (26.0), 111 (44.0), 124 (18.0), 125 (17.0), 143 (30.0), 152 (61.0), 153 (7.0), 185 (31.0)<br>41, 42, 43, 55, 56, 57, 59, 67, 68, 69,73, 74, 81, 82, 83, 84, 96, 97, 111, 124, 125, 143, 152, 153, 185 standard from the library NIST
M125	30.22	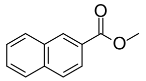	74 (11.0), 75 (12.0), 77 (17.0), 126 (21.0), 127 (100.0), 128 (8.0), 155 (90.0), 156 (13.0), 186 (48.0)<br>74, 75, 77, 126, 127, 128, 155, 156, 186 standard from the library NIST
M125<br>a	30.23	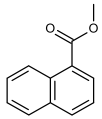	63 (15), 74 (13.0), 75 (14.0), 77 (25.0), 126 (24.0), 127 (100.0), 128 (12.0), 155 (91.0), 156 (13.0), 186 (57.0)<br>63, 74, 75, 77, 126, 127, 128, 155, 156, 186 standard from the library NIST
M126	32.6<br>32.4 st	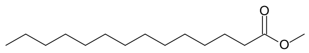<br>C14	41 (26.0), 43 (31.0), 55 (23.0), 57 (11.0), 59 (11.0), 69 (7.0), 74 (100.0), 75 (13.0), 87 (61.0), 143 (15.0), 199 (9.0), 242 (4.0)<br>41 (21.0), 43 (25.0), 55 (23.0), 57 (11.0), 59 (9.0), 69 (12.0), 74 (100.0), 75 (16.0), 87 (65.0), 143 (17.0), 199 (11.0), 242 (6.0) measured standard<br>41, 43, 55, 57, 59, 69, 74, 75, 87, 143, 199, 242 standard from the library NIST
M127	33.5	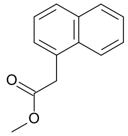	115 (42.0), 139 (26.0), 141 (100.0), 200 (53.0)<br>115, 139, 141, 200 standard from the library NIST
M128	34.5	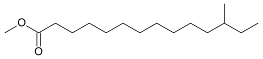	41(26.0), 55 (40.0), 56 (7.0), 57 (12.0), 67 (6.0), 74 (100.0), 75 (17.0), 83 (12.0), 87 (64.0), 97 (10.0), 143 (13.0), 177 (4.0), 199 (16.0), 227 (4.0), 256 (4.0)<br>41, 55, 56. 57, 67, 74, 75, 83, 87, 97, 143, 177 199, 227, 256 standard from the library NIST
M129	35.7<br>36.0 st	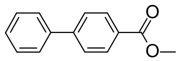	51 (5.0), 76 (28.0), 102 (8.0),126 (10.0), 127 (5.0), 151 (16.0), 152 (56.0), 153 (35.0), 181 (100.0), 182 (36.0), 212 (63.0)<br>51 (4.0), 76 (20.0), 102 (3.0), 126 (5.0), 127 (5.0), 151 (16.0), 152 (58.0), 153 (30.0), 181 (100.0), 182 (14.0), 212 (61.0) measured standard<br>51, 76, 102, 126, 127, 151, 152, 153, 181, 182, 212 standard from the library NIST

Supplementary Table S8. Mass spectrometric data of aliphatic and aromatic acids identified during growth on crude oil of *Micrococcus luteus* SBUG 2286. These compounds were analysed by GC/MS. The extracted acids were transformed for analytical purposes by methylation to the corresponding methyl esters, some acids occur in unmethylated form.

**No**	**t_R (min)**	**Metabolites**	**m/z (%) (70 eV) - analyzed with GC/MS**
M130	4.9	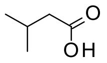	39 (12.0), 41 (46.0), 42 (15.0), 43 (47.0), 45 (25.0), 60 (100.0), 87 (18.0)<br>39, 41, 42, 43, 45, 60, 87 standard from the library NIST
M131	5.1	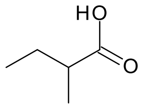	39 (33.0), 41 (47.0), 55 (17.0), 57 (63.0), 73 (14.0), 74 (100.0), 87 (21.0)<br>39, 41, 55, 57, 73, 74, 87 standard from the library NIST
M132	7.8	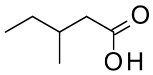	41 (35.0), 43 (18.0), 45 (21.0), 56 (17.0), 57 (18.0), 60 (100.0), 61 (14.0), 87 (18.0)<br>41, 43, 45, 56, 57, 60, 61, 87 standard from the library NIST
M133	10.8	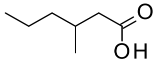	41 (42.0), 42 (22.0), 43 (51.0), 45 (22.0), 55 (28.0), 60 (100.0), 61 (21.0), 69 (9.0), 70 (28.0), 71 (23.0), 73 (16.0), 87 (32.0), 101 (1.0), 115 (3.0), 129 (0.0)<br>41, 42, 43, 45, 55, 60, 61, 69, 70, 71, 73, 101, 115, 129 standard from the library NIST
M134	11.5	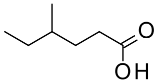	41 (67.0), 42 (14.0), 43 (51.0), 45 (26.0), 55 (77.0), 56 (18.0), 57 (35.0), 60 (36.0), 61 (4.0), 69 (18.0), 71 (100.0), 73 (65.0), 74 (40.0), 83 (30.0), 101 (20.0)<br>41, 42, 43, 45, 55, 56, 57, 59, 60, 61, 69, 71, 73, 74, 83, 101 standard from the library NIST
M135	13.4<br>13.6 st	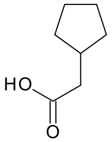	39 (29.0), 41 (51.0), 42 (32.0), 43 (34.0), 55 (50.0), 60 (100.0), 61 (64.0), 67 (26.0), 68 (58.0), 69 (36.0), 85 (6.0), 99 (12.0), 128 (4.0)<br>39 (26.0), 41 (46.0), 42 (15.0), 43 (11.0), 55 (26.0), 60 (100.0), 61 (64.0), 67 (31.0), 68 (66.0), 69 (33.0), 85 (27.0), 99 (20.0), 128 (3.0) measured standard<br>39, 41, 42, 43, 55, 60, 6, 67, 68, 69, 85, 99, 128 standard from the library NIST
M136	13.92	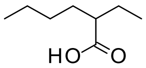	41 (22.0), 57 (32.0), 73 (83.0), 87 (20.0), 88 (100.0), 101 (12.0), 115 (10.0), 116 (13.0)<br>41, 57, 73, 87, 88, 101, 115, 116 standard from the library NIST
M137	14.7	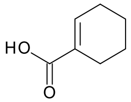	39 (26.0), 52 (8.0), 79 (59.0), 80 (8.0), 81 (100.0), 125 (7.0)<br>39, 52, 79, 80, 81, 125 standard from the library NIST

M138	15.35	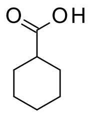	50 (30.0), 51 (45.0), 52 (12.0), 76 (9.0), 77 (100.0), 105 (93.0), 106 (10.0), 122 (77.0), 123 (8.0)<br>50, 51, 52, 76, 77, 105, 106, 122, 123 standard from the library NIST
M139	15.8	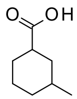	39 (28.0), 41 (41.0), 42 (10.0), 43 (15.0), 53 (13.0), 54 (10.0), 55 (100.0), 56 (11.0), 67 (10.0), 69 (17.0), 70 (43.0), 73 (17.0), 83 (18.0), 127 (10.0), 142 (17.0)<br>39, 41, 42, 43, 53, 54, 55, 56, 67 , 69, 70, 73, 83, 127, 142 standard from the library NIST
M140	16.8	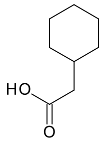	39 (45.0), 41 (67.0), 54 (25.0), 55 (98.0), 60 (100.0), 61 (56.0), 67 (66.0), 81 (53.0), 82 (73.0), 83 (76.0), 142 (4.0)<br>39, 41, 54, 55, 60, 61, 67, 81, 82, 83, 142 standard from the library NIST
M141	17.6	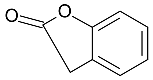	38 (10.0), 39 (17.0), 50 (16.0), 52 (21.0), 77 (19,0), 78 (100.0), 106 (36.0), 134 (45.0)<br>38, 39, 50, 51, 52, 77, 78, 106, 134 standard from the library NIST
M142	18.0	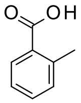	39 (32.0), 50 (13.0), 51 (12.0), 63 (34.0), 65 (44.0), 89 (27.0), 90 (87.0), 91 (100.0), 118 (87.0), 119 (33.0), 136 (78.0)<br>39, 50, 51, 63, 65, 89, 90, 91, 118, 119, 136 standard from the library NIST
M143	18.3	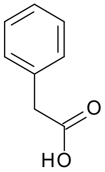	51 (7.0), 65 (18.0), 91 (100.0), 92 (17.0), 136 (24.0)<br>51, 65, 91, 92, 136 standard from the library NIST
M144	18.6	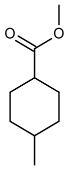	39 (20.0), 41 (28.0), 54 (7.0), 55 (59.0), 60 (36.0), 61 (18.0), 67 (20.0), 70 (), 81 (40.0), 82 (2.0), 87 (2.0), 95 (5.0), 96 (22.0), 97 (100.0), 113 (), 124 (1.0), 125 (1.0), 141 (), 156 (1.0)<br>39, 41, 54, 55, 60, 61, 67, 70, 81, 82, 87, 95, 96, 97, 98, 113, 124, 125, 141, 156 standard from the library NIST

M145	18.9	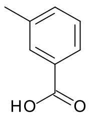	38 (9.0), 62 (5.0), 63 (15.0), 65 (28.0), 89 (8.0), 90 (7.0), 91 (100.0), 92 (17.0), 119 (52.0), 120 (4.0), 136 (49.0), 137 (6.0)<br>38, 62, 63, 65, 89, 90, 91, 92, 119, 120, 136, 137 standard from the library NIST
M146	19.10	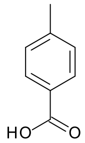	39 (16.0), 65 (22.0), 89 (14.0), 91 (100.0), 119 (58.0), 136 (50.0)<br>39 , 65, 89, 91, 119, 136 standard from the library NIST
M147	19.12<br>19.4 st	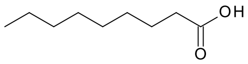 C9	39 (39.0), 41 (70.0), 42 (18.0), 43 (47.0), 45 (24.0), 55 (51.0), 56 (14.0), 57 (51.0), 60 (100.0), 61 (17.0), 87 (21.0), 98 (16.0), 115 (27.0), 129 (19.0), 158 (1.0)<br>39 (13.0), 41 (38.0), 42 (11.0), 43 (31.0), 45 (13.0), 55 (37.0), 56 (12.0), 57 (56.0), 60 (100.0), 61 (13.0), 87 (14.0), 98 (16.0), 115 (33.0), 129 (28.0), 158 (2.0) measured standard<br>39, 41, 42, 43, 45, 55, 56, 57, 60, 61, 87, 98, 115, 129, 158 standard from the library NIST
M148	20.3	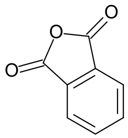	38 (12.0), 50 (52.0), 74 (25.0), 76 (94.0), 104 (100.0), 148 (16.0)<br>38, 50, 74, 76, 104, 148 standard from the library NIST
M149	21.4	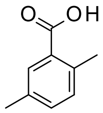	50 (13.0), 51 (18.0), 52 (6.0), 77 (50.0), 78 (21.0), 103 (21.0), 104 (85.0), 105 (100.0), 132 (65.0), 133 (9.0), 150 (51.0)<br>50, 51, 52, 77, 78, 103, 104, 105, 132, 133, 150 standard from the library NIST
M150	21.5	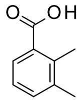	39 (18.0), 77 (32.0), 78 (21.0), 79 (32.0), 103 (35.0), 104 (55.0), 105 (100.0), 132 (84.0), 133 (30.0), 150 (68.0)<br>39, 77, 78, 79, 103, 104, 105, 132, 133, 150 standard from the library NIST
M151	21.7	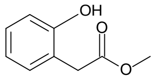	39 (16.0), 50 (12.0), 51 (46.0), 52 (25.0), 53 (18.0), 62 (5.0), 63 (11.0), 77 (100.0), 78 (87.0), 79 (42.0), 105 (71.0), 106 (90.0), 107 (78.0), 134 (65.0), 135 (67.0), 166 (29.0)<br>39, 50, 51, 52, 53, 62, 63, 77, 78, 79, 105, 106, 107, 134, 135, 166 standard from the library NIST
M152	22.1	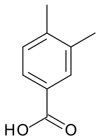	51 (16.0), 77 (33.0), 78 (17.0), 79 (17.0), 90 (6.0), 103 (15.0), 105 (100.0), 132 (8.0), 150 (75.0)<br>51, 77, 78, 79, 90, 103, 105, 132, 150 standard from the library NIST

M153	24.1	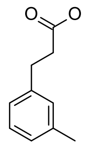	39 (30.0), 41 (38.0), 52 (5.0), 77 (25.0), 78 (8.0), 79 (15.0), 91 (31.0), 92 (8.0), 105 (100.0), 119 (24.0), 164 (38.0)<br>39, 41, 52, 77, 78, 79, 91, 92, 105, 119, 164 standard from the library NIST
M154	24.3	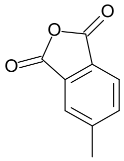	39 (10.0), 62 (15.0), 63 (28.0), 89 (58.0), 90 (65.0), 118 (100.0), 162 (20.0)<br>39, 62, 63, 89, 90, 118, 162 standard from the library NIST
M155	40.0	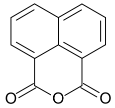	63 (26.0), 73 (9.0), 74 (16.0), 126 (95.0), 154 (100.0), 155 (10.0), 198 (38.0)<br>63, 73, 74, 126, 154, 155, 198 standard from the library NIST

Supplementary Table S9. Mass spectrometric data of aliphatic and aromatic acids identified during growth on crude oil of *Bacillus atrophaeus* SBUG 2291. These compounds were analysed by GC/MS. The extracted acids were transformed for analytical purposes by methylation to the corresponding methyl esters, some acids occur in unmethylated form.

No	t_R (min)	Metabolites	m/z (%) (70 eV) - analyzed with GC/MS
M156	12.8<br>13.1 st	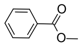	50 (11.0), 51 (29.0), 77 (67.0), 92 (7.0), 105 (100.0), 106 (9.0), 136 (34.0)<br>50 (7.0), 51 (17.0), 77 (56.0), 92 (2.0), 105 (100.0), 106 (8.0), 136 (38.0) measured standard<br>50, 51, 77, 92, 105, 106, 136 standard from the library NIST
M157	14.8<br>15.1 st	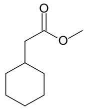	55 (33.0), 67 (12.0), 74 (100.0), 75 (37.0), 81 (12.0), 97 (19.0), 113 (3.0), 125 (5.0), 156 (1.0)<br>55 (16.0), 67 (7.0), 74 (100.0), 75 (34.0), 81 (6.0), 97 (6.0), 113 (2.0), 125 (9.0), 156 (1.0) measured standard<br>55, 67, 74, 75, 8, 97, 113, 125, 156 standard from the library NIST
M158	15.7<br>16.1 st	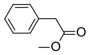	39 (7.0), 51 (4.0), 59 (6.0), 65 (15.0), 77 (2.0), 89 (7.0), 91 (100.0), 92 (8.0), 105 (1.0), 119 (2.0), 150 (22.0)<br>39 (4.0), 51 (2.0), 59 (5.0), 65 (12.0), 77 (1.0), 89 (5.0), 91 (100.0), 92 (8.0), 105 (1.0), 119 (2.0), 150 (34.0) measured standard<br>39, 51, 59, 65, 77, 89, 91, 92, 105, 119, 150 standard from the library NIST
M159	16.9	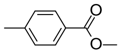	39 (8.0), 63 (11.0), 65 (17.0), 91 (43.0), 119 (100.0), 120 (9.0), 150 (21.0)<br>39, 63, 65, 91, 119, 120, 150 standard from the library NIST
M160	17.4<br>17.6 st	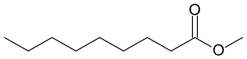 C9	39 (3.0), 41 (15.0), 55 (28.0), 59 (19.0), 74 (100.0), 75 (10.0), 83 (5.0), 87 (50.0), 88 (15.0), 101 (9.0), 129 (10.0), 141 (7.0), 143 (6.0), 172 (2.0)<br>39 (5.0), 41 (15.0), 55 (17.0), 59 (11.0), 74 (100.0), 75 (8.0), 83 (3.0), 87 (48.0), 88 (4.0), 101 (6.0),129 (11.0), 141 (11.0), 143 (9.0), 172 (1.0) measured standard<br>39, 41, 55, 59, 74, 75, 83, 87, 88, 101, 129, 141, 143, 172 standard from the library NIST
M161	17.5	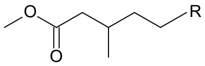	41 (47.0), 43 (48.0), 57 (37.0), 59 (17.0), 73 (6.0), 74 (100.0), 75 (16.0), 101 (70.0)<br>41, 43, 57, 59, 73, 74, 75, 101 structure deduced from fragmentation and retention time
M162	19.8	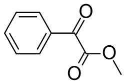	50 (10.0), 51 (23.0), 77 (62.0), 105 (100.0), 106 (7.0), 164 (2.0)<br>50, 51, 77, 105, 106, 164 standard from the library NIST
M163	21.4	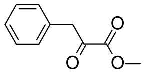	65 (13.0), 89 (6.0), 90 (6.0), 91 (100.0), 118 (12.0), 119 (8.0), 178 (8.0)<br>65, 89, 90, 91, 118, 119, 178 standard from the library NIST

M164	32.7<br>32.4 st	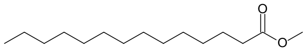 C14	41 (18.0), 43 (14.0), 55 (19.0), 59 (11.0), 69 (25.0), 74 (100.0), 75 (14.0), 87 (61.0), 143 (15.0), 199 (9.0), 242 (5.0)<br>41 (21.0), 43 (25.0), 55 (23.0), 59 (9.0), 69 (12.0), 74 (100.0), 75 (16.0), 87 (65.0), 143 (17.0), 199 (11.0), 242 (6.0) measured standard<br>41, 43, 55, 59, 69, 74, 75, 87, 143, 199, 242 standard from the library NIST
M165	34.5<br>34.3st	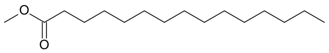 C15	74 (100.0), 75 (17.0), 87 (66.0), 143 (15.0), 213 (10.0), 256 (4.0)<br>74 (100.0), 75 (17.0), 87 (71.0), 143 (20.0), 213 (14.0), 256 (11.0) measured standard<br>74, 75, 87, 143, 213, 256 standard from the library NIST
M166	44.6	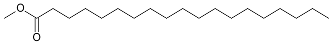 C19	59 (9.0), 74 (100.0), 75 (25.0), 87 (68.0), 88 (9.0), 143 (24.0), 269 (5.0), 312 (7.0)<br>59, 74, 75, 87, 88, 143, 269, 312 standard from the library NIST
M167	46.7	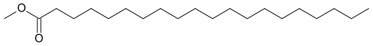 C20	59 (9.0), 74 (100.0), 75 (26), 87 (73.0), 143 (19.0), 283 (7.0), 326 (12.0)<br>59, 74, 75, 87, 143, 283, 326 standard from the library NIST
M168	48.7	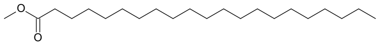 C21	74 (100.0), 75 (26), 87 (81.0), 143 (17.0), 241 (4.0), 297 (10.0), 340 (13.0)<br>74, 75, 87, 143, 241, 297, 340 standard from the library NIST
M169	50.7	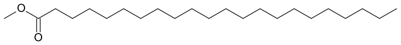 C22	69 (11.0), 71 (13.0), 74 (100.0), 75 (28.0), 87 (70.0), 111 (3.0), 143 (21.0), 255 (6.0), 311 (6.0), 354 (13.0)<br>69, 71, 74, 75, 87, 111, 143, 255, 311, 354 standard from the library NIST
M170	52.6	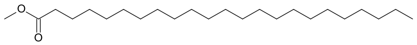 C23	42 (13.0), 43 (23.0), 55 (10.0), 71 (16.0), 74 (89.0), 75 (30.0), 87 (76.0), 143 (25.0), 269 (5.0), 325 (11.0), 368 (19.0)<br>42, 43, 55, 71, 74, 75, 87, 143, 269, 325, 368 standard from the library NIST
